# A Cell-Based Systems Biology Assessment of Human Blood to Monitor Immune Responses after Influenza Vaccination

**DOI:** 10.1371/journal.pone.0118528

**Published:** 2015-02-23

**Authors:** Kristen L. Hoek, Parimal Samir, Leigh M. Howard, Xinnan Niu, Nripesh Prasad, Allison Galassie, Qi Liu, Tara M. Allos, Kyle A. Floyd, Yan Guo, Yu Shyr, Shawn E. Levy, Sebastian Joyce, Kathryn M. Edwards, Andrew J. Link

**Affiliations:** 1 Department of Pathology, Microbiology and Immunology, Vanderbilt University School of Medicine, Nashville, TN, 37232, United States of America; 2 Department of Biochemistry, Vanderbilt University School of Medicine, Nashville, TN, 37232, United States of America; 3 Vanderbilt Vaccine Research Program; Division of Infectious Diseases, Department of Pediatrics, Vanderbilt University School of Medicine, Nashville, TN, 37232, United States of America; 4 HudsonAlpha Institute for Biotechnology, Huntsville, AL, 35806, United States of America; 5 Department of Chemistry, Vanderbilt University, Nashville, TN, 27232, United States of America; 6 Department of Biomedical Informatics, Vanderbilt University School of Medicine, Nashville, TN, 37232, United States of America; 7 Department of Cancer Biology, Vanderbilt University School of Medicine, Nashville, TN, 37232, United States of America; 8 Department of Cancer Biostatistics, Vanderbilt University School of Medicine, Nashville, TN, 37232, United States of America; Massachusetts General Hospital, UNITED STATES

## Abstract

Systems biology is an approach to comprehensively study complex interactions within a biological system. Most published systems vaccinology studies have utilized whole blood or peripheral blood mononuclear cells (PBMC) to monitor the immune response after vaccination. Because human blood is comprised of multiple hematopoietic cell types, the potential for masking responses of under-represented cell populations is increased when analyzing whole blood or PBMC. To investigate the contribution of individual cell types to the immune response after vaccination, we established a rapid and efficient method to purify human T and B cells, natural killer (NK) cells, myeloid dendritic cells (mDC), monocytes, and neutrophils from fresh venous blood. Purified cells were fractionated and processed in a single day. RNA-Seq and quantitative shotgun proteomics were performed to determine expression profiles for each cell type prior to and after inactivated seasonal influenza vaccination. Our results show that transcriptomic and proteomic profiles generated from purified immune cells differ significantly from PBMC. Differential expression analysis for each immune cell type also shows unique transcriptomic and proteomic expression profiles as well as changing biological networks at early time points after vaccination. This cell type-specific information provides a more comprehensive approach to monitor vaccine responses.

## Introduction

Systems biology is a comprehensive approach to describe complex interactions between multiple components in a biological system[1]. Using high-dimensional molecular approaches, systems biology identifies changes caused by perturbations such as infection or vaccination, combined with extensive computational analysis to model and predict responses[2,3]. In the context of vaccinology, systems biology offers an approach to dissect the human immune response after immunization by correlating changes in the transcriptome and proteome with antibody or cell-mediated immune responses, in order to make predictions about vaccine efficacy and potentially adverse events[4,5].

The first systems biological studies to dissect human vaccine-induced responses utilized the yellow fever vaccine, YF-17D[6,7]. In these pioneering studies, both CD8+ T cell and B cell signatures identified in microarray profiles were correlated with protective cell-mediated and antibody responses, thus providing predictive signatures. Since these studies, several other vaccines have been studied, including live and inactivated influenza and pneumococcal polysaccharide vaccines [8–10]. Systems biology studies with influenza vaccines identified modules of genes that positively correlated with protective immune responses. For example, interferon-responsive genes that were up-regulated at early time points after TIV vaccination positively correlated with robust hemagglutinin inhibition (HAI) titers[8,10]. Nakaya *et al*. found that an elevated antibody response to trivalent inactivated influenza vaccine (TIV), but not to live attenuated influenza vaccine (LAIV), correlated with upregulation of B cell-specific transcripts, including immunoglobulins (IgA, IgD, IgE, and multiple IgGs) and the TNFRSF17 surface receptor[9]. Using the Nakaya dataset, Tan *et al*. identified immunoglobulin and complement genes as well as proliferation-associated genes to be predictors of protective antibody production in response to TIV vaccination. They concluded that enrichment of these particular gene sets at 7 days post-TIV vaccination was likely due to increased representation of proliferating plasmablasts in subjects with elevated antibody responses[11].

Predictive correlates that can be identified prior to vaccination are emerging in systems vaccinology studies. Tsang *et al*. recently showed that baseline proportions of 126 individual immune cell sub-populations in the blood, identified by comprehensive flow cytometric analysis, could predict influenza vaccine-induced antibody responses[12]. Several studies have found an inverse correlation between baseline influenza-specific microneutralization or HAI titers and the subsequent generation of both plasmablasts and protective antibodies after seasonal influenza vaccination. These studies reported that subjects with lower baseline titers of influenza antibodies generated more robust post-vaccine antibody responses compared to subjects with high baseline titers [12,13]. Furman *et al*. identified several additional baseline predictors of protective immunity, including the frequency of CD8 T cells and NK cells, as well as multiple differentially expressed gene modules. These included genes associated with: 1) apoptotic pathways; 2) cell survival and proliferation (including generation and maintenance of germinal centers); 3) cell-to-cell signaling; 4) RNA post-transcriptional modification; and 5) carbohydrate metabolism [13].

Despite insights into the global human immune responses obtained from these and other studies, the majority of systems biology studies are limited in scope to total RNA from whole blood or peripheral blood mononuclear cells (PBMC)[6–8,11–18]. Since human blood is comprised of a multitude of hematopoietic cell types that are present in varying proportions, responses elicited from under-represented cell types in the blood are likely masked by those of predominant cells[19]. For example, Nakaya *et al*. found upregulation of the transcription factor XBP-1, which is necessary for the terminal differentiation of antibody-forming plasma cells, in RNA from sorted B cells, but not from PBMC, after TIV vaccination[9,20]. Additionally, when utilizing PBMC to monitor the immune response, the contributions of polymorphonuclear (PMN) cells—prime contributors to innate immunity—are overlooked.

Most current vaccines target adaptive immune T and B lymphocytes by conferring lasting, life-long immunity (memory) that can be recalled rapidly upon subsequent encounter with the immunizing antigen[21,22]. The qualitative and quantitative aspects of these adaptive immune responses are slow to develop and are tightly regulated by the rapidly-induced innate immune response. Thus, an immune response represents a highly coordinated effort from multiple hematopoietic cell types—each with their own inherent programming. We therefore believe that it is vitally important to analyze and model individual cell types in response to vaccination.

To develop a comprehensive systems biology model for studying immune responses following vaccination, we developed an efficient protocol to purify from human blood six immune cell types that contribute to both innate and adaptive immune responses: T cells, B cells, natural killer (NK) cells, myeloid dendritic cells (mDC), monocytes, and neutrophils. These cells were isolated and processed immediately for down-stream systems analysis to avoid potential problems associated with the use of frozen cells[23]. Unlike previous systems vaccinology studies, which utilized microarray analysis to map dynamic changes in the transcriptome after vaccination[6–10], this study utilized RNA-Seq data generated prior to and after TIV vaccination together with the human reference genome sequence to identify changes in both protein-coding and non-coding RNA transcripts after vaccination. Additionally, and unique to this study, our protocol included quantitative proteomics to monitor changes in protein expression after vaccination.

Our results reveal that RNA and protein expression profiles from each sorted cell type differ significantly from the profile obtained from PBMC. Comparison of differentially expressed transcripts and proteins after vaccination with 2011–2012 seasonal TIV further shows considerable differences between PBMC and sorted cells. Together, our data suggest that important cell type-specific information is gained when purified cells rather than PBMC or whole blood are utilized in systems studies. The cell type-specific information obtained from unbiased RNA-seq and quantitative proteomics analysis utilizing the complete human reference genome sequence provides a more comprehensive systems biology approach to monitor and eventually to model vaccine responses. This approach is applicable for other systems biology studies involving complex interactions between different cell types following vaccinations, infectious diseases, diseases and pharmacological interventions.

## Materials and Methods

### Seasonal TIV Vaccination of human volunteers and blood collection

Volunteer recruitment and vaccination protocols for this study were approved by the Vanderbilt Institutional Review Board (IRB#111030 “CLR-03 2011-Immune Cells and Soluble Factors from Healthy Donor”). After obtaining written informed consent, thirty one subjects were enrolled in this study. Twenty-nine subjects provided 90mL blood samples to develop our phenotyping and cell sorting protocols and to establish baseline blood profiling information; for these purposes, twenty three subjects provided a single blood sample, and six subjects provided four samples over subsequent days on the same schedule as proposed for vaccinated subjects. Once the cell sorting pipeline was in place, two subjects were vaccinated with a single dose of 2011–2012 seasonal trivalent inactivated influenza vaccine (TIV) (strains included: A/California/7/09 (H1N1,), A/Perth /16/2009 (H3N2), and B/Brisbane/60/2008). Blood samples (90mL) from the two vaccinated subjects were processed prior to vaccination (day 0) and at days 1, 3, and 7 post-vaccination for downstream RNA-seq and quantitative proteomics analysis.

### Immune cell purification and flow cytometric analysis

PBMC and PMN were isolated from anti-coagulated (EDTA) whole blood via Ficoll-paque PLUS (GE Healthcare) separation. Residual RBCs were removed from the PMN fraction by ammonium-chloride-potassium (ACK) lysis (KD Medical). Single cell suspensions of PBMC or PMN were subjected to magnetic bead separation. T cells, monocytes, and neutrophils were enriched by positive selection using directly conjugated anti-CD3, anti-CD14, and anti-CD15 microbeads (Miltenyi Biotec), respectively. B cells were enriched by positive selection using anti-PE beads after staining with anti-CD19-PE antibody (Miltenyi Biotech) since directly conjugated CD19-microbeads interfered with subsequent anti-CD19 phenotypic staining. NK/mDC were enriched by negative selection using Streptavadin microbeads (Miltenyi Biotec) after staining with biotinylated anti-CD19 (clone HIB19), anti-CD15 (clone HI98), anti-CD14 (clone 61D3), and anti-CD3 (clone UCHT1) antibodies (eBioscience). MACS enriched cells were stained with 7-aminoactinomycin D (7-AAD), CD11c-FITC (clone B-ly6) CD15-APC (clone HI98) and CD56-PE-Cy7 (clone B159) (BD Biosciences), as well as CD19-PE (130-091-247), CD3-VioBlue (130-094-363), and CD14-VioGreen (130-096-875) (Miltenyi Biotec), and were subjected to FACS on a BD FACSAriaIII flow cytometer. Cell purity of ≥98% was confirmed by re-analysis on the FACSAriaIII after the sort. Whole blood, PBMC, PMN and pooled sorted cells were subjected to 9-color flow cytometric analysis (FCM) to assess phenotype and cellular activation at each time point using the same sorting markers as above, without 7-AAD, and with addition of CD86-PerCP-Cy5.5 (clone FUN-1), CD69-APC-Cy7 (clone FN50), and CD134-PE-Cy5 (clone ACT35) (BD Biosciences). The SPHERO Ultra Rainbow calibration kit (Spherotech; URCP-50-2K) was utilized to control for daily fluctuations in the detectors used for activation marker staining. FCM was performed on a BD LSRFortessa flow cytometer, and data was analyzed using the *FlowJo* software package (Tree Star).

### RNA expression analysis

Total RNA was extracted from PBMC and sorted immune cells (≤0.5x10^6^ cells) from the two TIV-vaccinated subjects using the automated Maxwell 16 magnetic particle processor and a Maxwell 16 LEV simply RNA kit (Promega Corp.). RNA was quantified by either a Qubit fluorometer (Life Technologies) or the Quant-iT RiboGreen RNA Assay (Life Technologies). To assess RNA integrity, total RNA was evaluated on a Bioanalyzer 2100 (Agilent Technologies). One hundred ng of total RNA with RIN values >7 was required for proceeding to downstream RNA-seq applications. Polyadenylated RNAs were isolated using NEBNext magnetic oligo d(T)_25_ beads. NEBNext mRNA Library Prep Reagent Set for Illumina (New England BioLabs Inc.) was used to prepare individually bar-coded next generation sequencing expression libraries. Library quality was assessed by Qubit 2.0 Fluorometer (Invitrogen), and library concentration was estimated by utilizing a DNA 1000 chip on an Agilent 2100 Bioanalyzer (Applied Biosystems). Accurate quantification of the prepared libraries for sequencing applications was determined using the qPCR-based KAPA Biosystems Library Quantification kit (Kapa Biosystems, Inc.). Each library was diluted to a final concentration of 12.5nM and pooled equimolar prior to clustering. Paired-End (PE) sequencing (25 million, 50-bp, paired-end reads) was performed using a 200 cycle TruSeq SBS HS v3 kit on an Illumina HiSeq2000 sequencer (Illumina, Inc.). Image analysis and base calling was performed using the standard Illumina Pipeline consisting of Real time Analysis (RTA) version v1.13. Raw reads were de-multiplexed using a bcl2fastq conversion software v1.8.3 (Illumina, Inc.) with default settings. Post-processing of the sequencing reads from RNA-seq experiments from each sample was performed as per HudsonAlpha’s unique in-house pipeline. Briefly, quality control checks on raw sequence data from each sample were performed using *FastQC* (Babraham Bioinformatics). Raw reads were mapped to the reference human genome hg19/GRCh37 using TopHat v1.4[24,25]. The alignment metrics of the mapped reads were estimated using *SAMtools* (S1 Dataset. RNA-seq quality control)[26]. Aligned reads were imported onto the commercial data analysis platform *AvadisNGS* v1.5 (Strand Life Sciences). After quality inspection, the aligned reads were filtered on the basis of read quality metrics where reads with a base quality score less than 30, alignment score less than 95, and mapping quality less than 40 were removed. Remaining reads were then filtered on the basis of their read statistics, where missing mates, translocated, unaligned and flipped reads were removed. The reads list was then filtered to remove duplicates. Samples were grouped and quantification of transcript abundance was performed on this final read list using Trimmed Means of M-values (TMM) as the normalization method [27]. Output data utilized for all subsequent comparisons was a normalized signal value generated by *AvadisNGS*. Resulting transcript lists were quality checked using *AvadisNGS* on a cell-type and donor basis across time points using comparative analysis; transcripts from the same cell type and donor required a correlation coefficient >0.9 to be accepted for further analysis (S1 Fig.. RNA quality control).

### Quantitative proteomic analysis

Protein extracts from PBMC and sorted immune cells (1x10^6^ cells) from the two vaccinated subjects were prepared as previously described[28] using a modified lysis buffer (50% Trifluoroethanol 50 mM HEPES) and quantified by BCA assay[29]. An immune cell common standard (ICCS) control sample composed of protein extracts from PBMC and CD15^+^ cells (80% and 20%, respectively, by protein weight) was included in all 8plex iTRAQ experiments. Ten ug of reduced, alkylated, and trypsinized protein extracts were labeled with iTRAQ tags (AB Sciex), pooled, and analyzed by MudPIT using an Eksigent 2-D nanoLC pump coupled to a nanoESI-LTQ-OrbitrapXL mass spectrometer (Thermo Scientific)[30,31]. The precursor ions were analyzed in the Orbitrap followed by 4 collision induced dissociation (CID) fragment ion scans in the ion trap to identify peptides. The precursor ions were then fragmented by higher-energy collisional dissociation (HCD) to measure reporter ion intensities in the Orbitrap. For each precursor ion, the CID and HCD spectra were merged using *Proteome Discoverer v1.3* (Thermo Scientific). The merged fragmentation spectra were searched against a forward and reverse concatenated human Ensembl protein and common contaminants database (gene model 74) using the *Sequest* database search engine running under *Proteome Discoverer* [32,33]. Precursor mass tolerance was set to 20 ppm and fragment mass tolerance was set to 0.8 Da. iTRAQ modification of N-terminus and ε-amine of lysines and β-methylthiolation of cysteines were used as static/constant modifications of the peptides. Oxidation of methionine and tryptophan and deamidation of asparagine and glutamine were used as dynamic/variable modifications of the peptides. Protein assembly, reporter ion quantitation and statistical analysis were performed with a 5% peptide and protein FDR using *ProteoIQ* v2.61 (Premier Biosoft). A slope of the regression line >0.8 between the technical replicates of the common control (ICCS) based upon pseudospectral counts was required as a quality control threshold (S2 Fig.. Proteomics quality control).

### Comparative and differential analysis

Comparative analysis of RNA transcripts and proteomics profiles between cell types was performed using Spearman correlation coefficients. Principal component analysis (PCA) was performed in R and plotted using the *rgl* package[34]. Heirarchical clustering analysis and dendograms were generated using *Cluster3.0* and *Java Treeview*, respectively [35,36]. Differential RNA transcript expression analysis was performed in *AvadisNGS v1.5*. RNA transcripts were first filtered to include only reads that met a threshold of 0.5 RPKM in at least one time point on a per-cell type and per-subject basis. Next, a Z-test (theoretical estimate of variance), in which the Benjamini-Hochberg procedure was used to fix the FDR at 0.05, was applied to pair-wise comparisons (days 0–1, 0–3, and 0–7) on a per cell-type and per-subject basis (*AvadisNSG v1.5*, Strand Life Sciences) [37]. Differential expression of transcripts was then calculated on the basis of fold change[38]. A ≥1.5 fold change in expression between time points was considered significant. Venn diagrams were used to identify differentially expressed transcripts between individuals and cell types. To identify potential differential splicing events in the RNA-Seq data, the publically accessible data analysis package *Multivariate Analysis of Transcript Splicing (MATS)* was used[39]. *MATS* uses a multivariate uniform distribution to model the between-sample correlation in exon splicing patterns, and a Markov chain Monte Carlo (MCMC) method coupled with a simulation-based adaptive sampling procedure to calculate the P value and false discovery rate (FDR) of differential alternative splicing. Transcripts expressing the same differential splice event with both a *p≤0.05* and FDR≤0.05 from both subjects were identifeid as significant. For differential protein expression analysis following vaccination, fold changes were calculated in *ProteoIQ*. A plot of log2 fold changes against pseudospectral counts was used to assess the effect of sampling over the observed fold changes. The symmetric distribution of log2 fold changes versus pseudospectral counts suggests the differential expression analysis was unbiased by protein abundances (S2 Fig.). Distribution of fold changes across different samples was visualized using cluster dot plots (S2 Fig.). Missing values and contaminating keratin proteins were removed prior to differential analysis. A ≥1.25 fold change in expression between pair-wise comparisons (days 0–1, 0–3, and 0–7) was considered significant. A Unix bash shell command was used to identify differentially expressed proteins shared between individuals and cell types, as well as to create lists of DE genes and proteins for heat maps. Heat maps of RNA and protein fold changes following vaccination were generated using *Cluster3.0* and *Java Treeview*.

### Visualization of RNA and proteins across the human genome

Genome-wide visualization of relative RNA or protein expression from PBMC and each purified immune cell type was generated using the open-source Circos software package[40]. The genome location for individual transcript and protein data points was mapped using *BioMart*[41].

### Network analysis

Differentially expressed protein-coding RNA transcripts and proteins identified in both subjects after vaccination were imported into *Ingenuity Pathway Analysis* (Qiagen) to identify the most significantly affected unique canonical pathways, biological functions and networks between time points.

## Results

### Immune cell isolation

**Fig 1:**
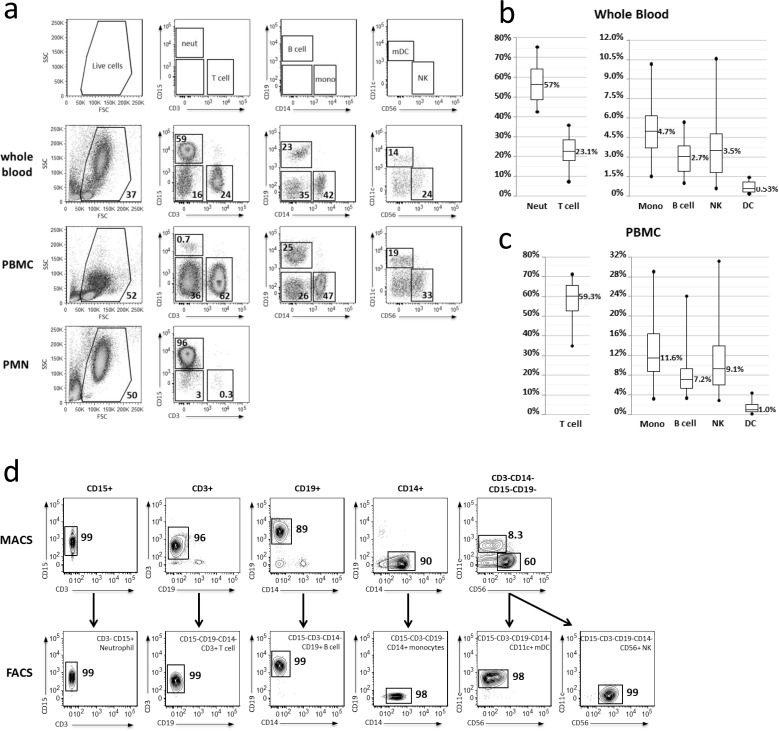
Flow cytometric analysis of immune cell types purified from human blood. **(a)** Whole blood (top panel), PBMC (middle panel) and PMN (bottom panel) cell samples from a single representative subject were stained with a cocktail of antibodies directed against CD3, CD11c, CD14, CD15, CD19, and CD56 cell surface markers for phenotypic analysis by flow cytometry. Moving left-to-right, live cells were first gated for CD3 and CD15 expression. Subsequent gates were drawn from the negative population in the previous panel. **(b,c)** Graphical representation of flow cytometric analysis from whole blood and the PBMC fraction reveals the variability among subjects (n = 31). **(d)** PMN and PBMC cell fractions from a single representative subject were subjected to CD15^+^, CD3^+^, CD19^+^, and CD14^+^ positive selection or CD19^-^CD15^-^CD14^-^CD3^-^ enrichment (top panels) via magnetic sorting (MACS). MACS-enriched cells were stained with the same cocktail of antibodies as in (a), with addition of 7AAD to exclude non-viable cells, and subjected to FACS (bottom panels) following the same gating scheme as in (a) to obtain highly purified neutrophil, T cell, B cell, monocyte, m DC, and NK populations for systems analysis.

To obtain purified human immune cells, PBMC and PMN were immediately fractionated from freshly collected venous blood over a Ficoll density gradient. Average numbers of PBMC and PMN obtained from 90mL of fresh blood were 232.9 ± 96.6x10^6^ and 113.1 ± 70.0x10^6^ (average ± SD), respectively. These cells were stained with a cocktail of antibodies to identify and quantify six targeted immune cell types: CD3+ T cells, CD14+ monocytes, CD15+ neutrophils, CD19+ B cells, CD11c+ mDC, and CD56+ NK cells (Fig. 1A). Distribution of leukocyte cell types in the whole blood (Fig. 1B) and PBMC fraction (Fig. 1C) fell within the expected, physiologically accepted range (whole blood: neutrophils, 25–80%; T cells, 10–30%; B cells, 1–9%; monocytes, 5–11%; NK, 1–8%; mDC 0–1%); however, variability was observed between subjects.

The number of cells needed for enrichment of each cell type, as well as the order of enrichment, depended upon both the total number of PBMC obtained and the individual’s phenotypic blood profile. The standard sorting protocol was designed for use when 150–300x10^6^ PBMC were obtained from 90mL fresh blood, which occurred in 24/39 (62%) samples (S3 Fig. Flow chart for immune cell purification). To account for variability in the abundance and composition of each donor’s cells, alternative sorting schemes were developed to maximize recovery of all cell types if larger or smaller numbers of PBMC were obtained from 90mL fresh blood, which occurred in 9/39 (23%) and 6/39 (15%) samples, respectively (S3 Fig.). Additionally, if a phenotypic blood profile varied widely from the average, or if recovery of a particular cell type was sub-optimal on the first visit, the proportion of PBMC or PMN fraction dedicated to enrichment of the affected cell-type(s) was altered accordingly in subsequent visits.

PBMC and PMN fractions were first subjected to magnetic-activated cell sorting (MACS) to positively select CD3+ T cells, CD14+ monocytes, CD15+ neutrophils, CD19+ B cells or negatively enrich for CD3-CD14-CD15-CD19- NK and mDC (Fig. 1D, top panels). However, cell yields and purity were inconsistent, rarely resulting in greater than 90% purity from any sample. Therefore, MACS-enriched cells were further subjected to fluorescence-activated cell sorting (FACS). Using the same antibody cocktail employed for phenotyping, with addition of (7-AAD) to exclude non-viable cells, neutrophils (CD3^-^CD15^+^), T cells (CD15^-^CD19^-^CD14^-^CD3^+^), B cells (CD15^-^CD3^-^CD14^-^CD19^+^), monocytes (CD15^-/lo^CD3^-^CD19^-^CD14^+^), mDC (CD15^-^CD3^-^CD19^-^CD14^-^CD56^-^CD11c^+^), and NK cells (CD15^-^CD3^-^CD19^-^CD14^-^CD11c^-^CD56^+^) were sorted with greater than 98% purity (Fig. 1D, bottom panels) in a short period of time; each sort generally took 30 min or less. Purified cells were not significantly activated by the sorting process, as assessed by flow cytometric analysis of size and scatter as well as surface staining for activation markers (S4 Fig. Individual cell types are not activated by the sorting process).

By employing this approach for sorting 6 immune cells types from fresh whole blood, we consistently obtained sufficient cells for both transcriptomic and proteomics analysis. After FACS purification, cells were immediately processed and frozen for downstream RNA (≤0.5x10^6^ cells) and protein (1x10^6^ cells) analyses. Greater than 1.5x10^6^ of each cell type was typically collected, except for mDC (Table 1). Recovery of sorted mDC was sufficient only for RNA analysis; proteomic analysis was not performed on this cell type.

**Table 1:** Recovery of purified immune cells.

	Starting quantity of PBMC* or PMN^#^ (mean ± SD x 10^6^)	Cell recovery after MACS + FACS (mean ± SD x 10^6^)	N
**T cell**	21.7 ± 3.3	2.0 ± 0.70	36
**B cell**	83.1 ± 20.7	1.7 ± 0.69	38
**Monocyte**	75.0 ± 20.7	2.9 ± 0.39	22
**mDC**	114.9 ± 38.2	0.41 ± 0.31	26
**NK**		1.9 ± 0.98	26
**Neutrophil**	31.6 ± 8.2	2.9 ± 0.35	16

*T cells, B cells, monocytes, mDC and NK cells were enriched/purified from the PBMC fraction

# Neutrophils were enriched/purified from the PMN fraction.

### Transcriptomic and Proteomic analysis in two TIV-vaccinated subjects

Previous systems biology approaches investigating yellow fever and influenza vaccine responses utilized microarray analysis to map the transcriptome after vaccination[6–10]. We used a more comprehensive, sensitive, quantitative and unbiased approach, next-generation RNA sequencing (RNA-Seq), which measures the RNA expression profile of each sample more accurately over a greater dynamic range than microarray-based technologies[42]. In addition to identification of expected coding sequences, RNA-seq allows for identification of non-coding transcripts, splice variants, sequence polymorphisms, and previously unannotated genes[43]. Additionally, the majority of systems vaccinology studies have focused solely on transcriptional analysis to map the immune response, with only selected proteins validated. We also used unbiased quantitative proteomics in addition to transcriptional data to analyze the immune response after vaccination.

A minimum of 100 ng total RNA of high quality (RIN greater than 7) was required for the construction of polyadenylated RNA-seq libraries. Sufficient RNA (250–700 ng total RNA) of good quality was obtained from 0.5x10^6^ PBMC and FACS-sorted T cells, B cells, NK, monocytes and neutrophils, as well as from 0.4–0.5x10^6^ FACS-sorted mDC (S5 Fig. Adequate RNA quality and quantity is obtained from sorted immune cells for RNA-seq applications). While sufficient quantity of RNA was obtained from 0.5x10^6^ neutrophils for our studies, these cells consistently yielded less RNA compared to other cell types, suggesting that additional sorted neutrophils should be collected in the future for downstream RNA applications.

**Fig 2:**
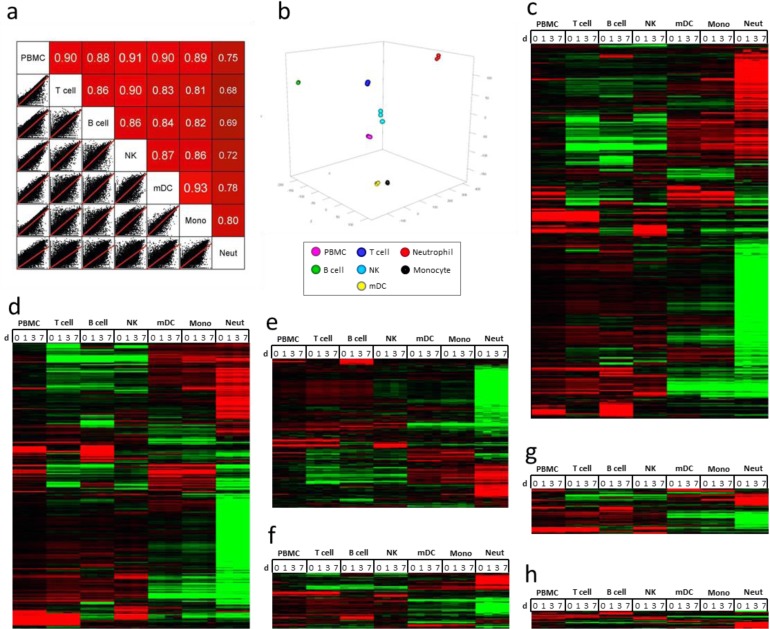
RNA-Seq analysis of purified immune cells after TIV vaccination. **(a)** Pair-wise comparison of day 0 RNA profiles (all transcript classes represented, filtered to remove zero values; 32,505 transcripts) from a vaccinated subject shows that the transcriptome of each sorted cell type correlates weakly with PBMC and other sorted cell types. **(b)** PCA of RNA profiles (all transcript classes represented, filtered to remove zero values; 37,606 transcripts) from a TIV-vaccinated subject at four time points shows that the purified immune cell types cluster into distinct groups, although monocytes and mDC cluster closely. **(c-h)** Semi-supervised hierarchical clustering analysis of RNA expression from a vaccinated individual reveals that purified immune cells have distinct RNA expression profiles compared to PBMC at all time-points. Data (non-zero transcripts with an RPKM of 1 in at least one sample) was centered for normalized signal value across gene and cell type; red = up, black = no change, green = down. **(c)** All transcript classes (21,438 transcripts). **(d)** Protein coding transcripts (13,243 transcripts, including Ig and TCR transcripts). **(e)** Pseudogenes (3,466 transcripts, 2x scale). **(f)** Anti-sense RNA (1,310 transcripts, 2x scale). **(g)** lincRNA (1,047 transcripts, 2x scale). **(h)** New genes (167 transcripts, 5x scale).

Using 25 million, 50-bp paired-end (PE) RNA-sequencing, the transcriptomes of PBMC as well as the six purified immune cell types from two subjects prior to (day 0) and at days 1, 3, and 7 after TIV vaccination were profiled. After the sequenced reads were aligned to the hg19 human reference genome and filtered to remove transcripts of poor quality, samples were loaded into *AvadisNGS v1.5* for downstream analysis. Approximately 56,000 transcripts were identified in 56 RNA samples (S2 Dataset. Normalized transcript expression in human immune cells prior to and post-TIV vaccination). Of these transcripts, 19,000–27,000 transcripts per cell type contained normalized signal values that were greater than zero. Twenty nine classes of RNA transcripts were identified, including protein coding RNA, pseudogenes, anti-sense RNA, long intervening non-coding RNA (lincRNA), and novel genes (S1 Table. Summary of baseline RNA transcripts identified in each cell type from one subject by RNA-seq analysis). Identification of non-polyA classes of RNA was likely caused by non-specific binding to oligo-dT or other inefficiencies during library construction; however, these classes constituted less than 2% of the total transcripts identified. Using Circos[40], PBMC and purified immune cell baseline (day 0) transcripts from a vaccinated subject plotted over the length of the human genome showed transcription was active across most of the genome, with small regions that appeared transcriptionally silent (S6 Fig. Transcriptional profiling of PBMC and individual immune cell types). Each of the purified immune cell types displayed distinct RNA expression profiles compared to PBMC and the other cell types. Pair-wise comparison of baseline (day 0) transcriptomes from the subject showed weak correlation between PBMC and each sorted cell type (Fig. 2A). Principal component analysis (PCA) of transcriptomes from each time point revealed that all cell types clustered distinctly based on RNA expression profiles (Fig. 2B). Finally, hierarchical clustering analysis of filtered transcripts revealed that each cell type displayed a distinct RNA expression profile that differed from both PBMC and the other cell types in all classes of RNA investigated (Fig. 2C-H) (S3 Dataset. Normalized transcript expression in human immune cells filtered for an RPKM of 1.0 in at least one sample from one subject).

Prior to performing quantitative proteomics, protein lysates were quantified. PBMC and sorted immune cells (1x10^6^) generated between 30–80 μg of protein/sample (S7 Fig. Adequate protein quantity is obtained from sorted immune cells for proteomics applications). In contrast to the RNA levels, neutrophils contained the highest amount of protein, while lymphocytes contained the least. Lysates from each sample were trypsinized, desalted, and labeled with 8plex iTRAQ reagents. A control sample—the Immune Cell Common Standard (ICCS)—was labeled with two iTRAQ channels to assess technical variation and used to normalize data across experiments. Two labeling strategies were tested to determine the optimal pooling strategy for detecting proteomic changes after vaccination (S8 Fig. Two iTRAQ strategies for quantitative proteomic analysis of immune cells after vaccination). In strategy 1, all six cell types at a single time point were multiplexed in one experiment. The advantage of this approach is that technical experimental variation between cell types at each time point would be minimized. However, since liquid chromatography tandem mass spectrometry (LC-MS/MS) selected proteins for identification and quantification based upon their abundance in the sample, proteins present in higher amounts across the samples would be preferentially quantified. Thus, differentially changing proteins with low expression from a single cell-type might not be quantified. Also, by increasing the complexity of the sample pool through multiplexing lysates from six different cell types, co-fragmentation of co-eluting peptides might cause an increase in iTRAQ signal interference. In strategy 2, all four time-points from one cell type were multiplexed in a single experiment. The advantage of this approach is that by pooling similar proteomes, sample complexity is reduced, thus reducing iTRAQ signal interference caused by co-fragmentation of co-eluting peptides. Since LC-MS/MS quantifies only a fraction of the proteome, this strategy would also ensure quantification of a larger fraction of cell type-specific proteins. However, cell type-specific changes that are artifacts might be detected due to technical experimental variation. We tested both strategies and analyzed the results using both unsupervised hierarchical clustering and PCA (S8 Fig.). Strategy 2 produced cell-type specific clustering and protein expression patterns by both hierarchical clustering and PCA, while strategy 1 did not. Since the samples in the iTRAQ experiments using strategy 1 did not cluster together by either hierarchical clustering or PCA, we discounted the possibility of batch effect. Therefore, strategy 2 was considered the optimal approach and employed for proteomic analysis.

**Fig 3:**
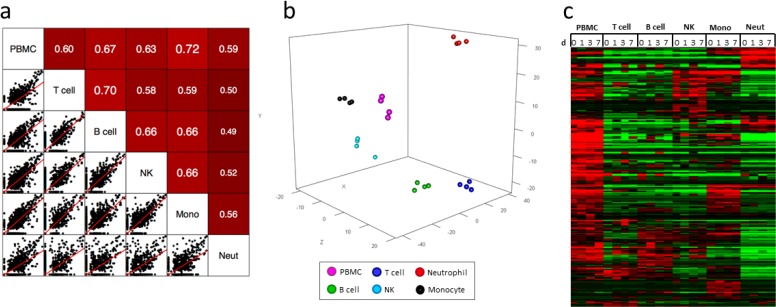
Proteomic analysis of purified immune cells after TIV vaccination. **(a)** Pair-wise comparison of day 0 protein profiles (3,852 proteins, filtered to remove zero values and contaminating keratins) from a vaccinated subject shows that proteomes of sorted cells correlate poorly with PBMC. **(b)** PCA of protein profiles from a TIV-vaccinated subject at four time points shows that purified immune cell types cluster into distinct groups. **(c)** Semi-supervised hierarchical clustering analysis of relative protein expression from a vaccinated individual reveals that purified immune cells have distinct proteomic expression profiles compared to PBMC. Data was centered across protein and cell type; red = up, black = no change, green = down.

Peptide spectra generated by LC-MS/MS were searched against the human Ensembl database of protein sequences using *Sequest* [33], and the resulting peptides were scored and assembled into proteins and quantified based upon the iTRAQ reporter ion intensities in *ProteoIQ*. The proteomes of PBMC and five purified immune cell types from two subjects prior to (day 0) and at days 1, 3, and 7 after TIV vaccination were analyzed. Approximately 7,000 proteins were identified in 44 protein samples (S4 Dataset. Normalized protein expression in human immune cells prior to and post-TIV vaccination). After removing zero values and contaminating keratins, approximately 4,000 proteins from each subject were retained for further analysis (S5 Dataset. Normalized protein expression in human immune cells filtered to remove zero values and contaminating keratins from one subject). Similar to transcriptomic analysis, the PBMC and purified immune cell baseline (day 0) proteomes from a vaccinated subject plotted over the length of the human genome showed activity across the majority of the genome (S9 Fig. Proteomic profiling of PBMC and individual immune cell types). Additionally, each of the purified immune cell types displayed distinct proteomic profiles when compared to PBMC and the other cell types. Pair-wise comparison of baseline (day 0) proteomic data from the subject showed poor correlation between PBMC and sorted cell types (Fig. 3A). PCA of proteomic data from each time point revealed that all cell types clustered distinctly based on proteomic profiles (Fig. 3B). Hierarchical clustering analysis of proteins identified showed that each cell type displayed a distinct protein expression profile that differed from both PBMC and the other cell types (Fig. 3C).

Strikingly, when clustering samples from both subjects in the same experiment by PCA, cell types from both subjects at every time point clustered similarly for RNA expression (∼39,000 transcripts, filtered to remove zero values). However, when analyzing protein data from both subjects (∼5,300 proteins, filtered to remove zero values and contaminating keratins), samples of the same cell type at every time point clustered similarly on a per-subject basis, but cells from the two subjects did not cluster together (S10 Fig. Principal component analysis reveals poor correlation of proteomes between subjects).

### Differential analysis of RNA and proteins from two TIV-vaccinated subjects

**Fig 4:**
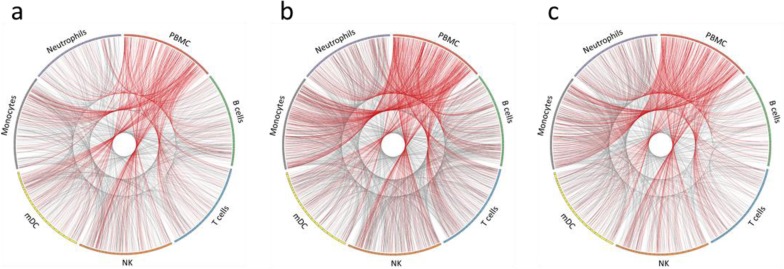
Visualization of differentially expressed RNA transcripts in PBMC and individual immune cell types. Circos plots of differentially expressed RNA transcripts from a vaccinated subject at **(a)** day 1, **(b)** day 3, and **(c)** day 7 post-TIV vaccination (fold change of ≥1.5x and p ≤ 0.05). All RNA transcript classes are represented. For each cell type, the colored bar on the outer circle represents the entire human genome; segments within the bars divide the genome into chromosomes. Red lines indicate DE transcripts that are shared between PBMC and purified immune cell types. Gray lines indicate DE transcripts that are shared between the purified immune cell types.

**Fig 5:**
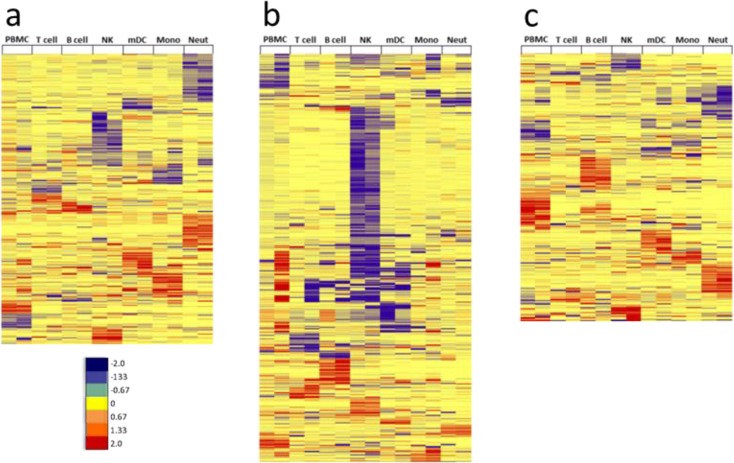
Unique modules of RNA transcripts are differentially expressed in each immune cell type after TIV vaccination. Differentially expressed RNA transcripts (≥1.5-fold change, p < 0.05) that were shared between both subjects after TIV-vaccination were subjected to semi-supervised hierarchical clustering analysis. Log2 fold-change values of shared DE transcripts in all cell types from both subjects were clustered at **(a)** day 1 (463 transcripts), **(b)** day 3 (653 transcripts), and **(c)** day 7 (428 transcripts) post-vaccination. Very little overlap of shared differentially expressed RNA transcripts is observed between cell types; red = up; yellow = no change; blue = down.

For comparison of transcriptional changes in PBMC and sorted immune cells, transcripts that were differentially expressed (DE) ≥1.5-fold (*p ≤ 0.05*) after vaccination were investigated. While standard methods for determining fold change typically use a 2x fold-change, we found that using this threshold failed to identify significant numbers of shared DE transcripts between both subjects. We therefore tested several different fold-change values, ranging from 1.25x-1.75x. By lowering the threshold to 1.5x, we obtained more comprehensive lists of DE transcripts from each cell type that were shared between both donors at each time point. When DE transcripts from PBMC were compared to DE transcripts from each purified immune cell type, less than 10% similarity was typically observed (S2 Table. Comparison of differentially expressed RNA transcripts in PBMC and individual immune cell types). Circos was used to plot DE transcripts from PBMC and each purified immune cell type from a vaccinated subject over the length of the human genome and to visualize overlap of differentially expressed genes at three time points after TIV vaccination (day 1, day 3, and day 7) (Fig. 4). The plots showed a lack of substantial overlap in differential expression between PBMC and each purified immune cell type. Interestingly, the three time points showed changing patterns of overlapping expression for PBMC and each cell type after TIV vaccination. Substantial variability was also observed in the number of cell type-specific DE transcripts when making subject-to-subject comparisons, with less than 10% similarity between donors for most cell types and time points (S3 Table. Shared DE RNA transcripts; S6 Dataset. Shared up-regulated DE RNA transcripts; and S7 Dataset. Shared down-regulated DE RNA transcripts). To minimize background noise, we only considered DE transcripts from each cell type that were shared in both subjects after TIV vaccination in further downstream investigations. Using semi-supervised hierarchical clustering, little overlap in the significantly changing protein-coding RNA transcripts was observed between each cell type and at each time point after TIV vaccination (Fig. 5A-C).

Additionally, RNA-seq analysis provided a platform to investigate differential splicing events after TIV vaccination. Using the *Multivariate Analysis of Transcript Splicing (MATS)* data analysis package[39], splicing events were identified in each cell type from each subject (S4 Table. Total splicing events identified in each cell type). Differential splicing events (*p≤0.05* and FDR≤0.05) were then identified in each cell type from each individual (S5 Table. Differential splicing events identified in each subject, cell type and time point). Several splicing events shared between both subjects were identified (S6 Table. Shared differential splicing events).

**Fig 6:**
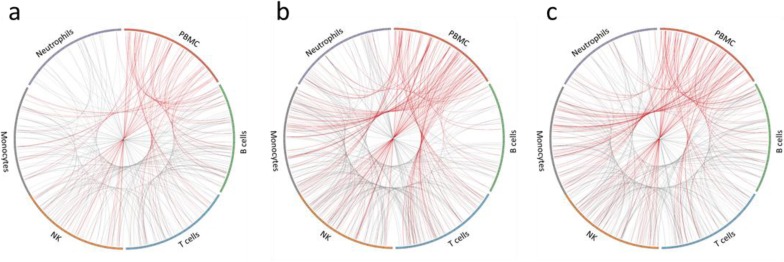
Visualization of differentially expressed proteins in PBMC and individual immune cell types. Circos plots of differentially expressed proteins from a vaccinated subject at **(a)** day 1, **(b)** day 3, and **(c)** day 7 post-TIV vaccination (fold change of ≥1.25x). For each cell type, the colored bar on the outer circle represents the entire human genome; segments within the bars divide the genome into chromosomes. Red lines indicate DE proteins that are shared between PBMC and purified immune cell types. Gray lines indicate DE proteins that are shared between the purified immune cell types.

**Fig 7:**
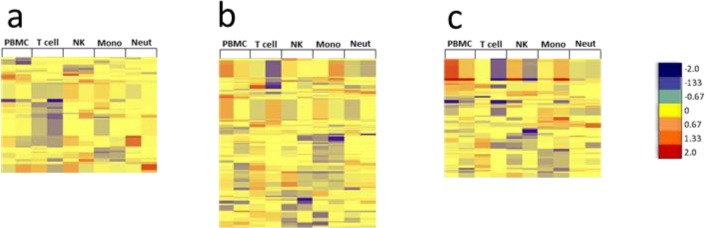
Unique modules of proteins are differentially expressed in each immune cell type after TIV vaccination. Differentially expressed proteins (≥1.25-fold change) that were shared between both subjects after vaccination with TIV were subjected to semi-supervised hierarchical clustering analysis. Log2 fold change values of shared DE proteins in each cell type from both subjects were clustered at **(a)** day 1 (196 proteins), **(b)** day 3 (263 proteins), and **(c)** day 7 (199 proteins) post-vaccination. Very little overlap of differentially expressed proteins is observed between cell types; red = up; yellow = no change; blue = down. B cell data was derived from only one subject due to insufficient recovery of B cells from the second subject.

For proteins, the DE threshold was lowered to ≥1.25-fold to adjust for iTRAQ under-reporting of fold changes[44]. By choosing this threshold, we obtained comprehensive lists of DE proteins from each cell type that were shared between both subjects at each time point. Similar to RNA, there was little correlation between PBMC and purified immune cell types when comparing DE proteins (S7 Table. Comparison of differentially expressed proteins in PBMC and individual immune cell types). Circos was used to plot DE proteins from PBMC and each purified immune cell type in a vaccinated subject over the length of the human genome and to visualize overlap of differentially expressed proteins at three time points after TIV vaccination (day 1, day 3, and day 7) (Fig. 6). Similar to RNA data, the plots showed a lack of substantial overlap in DE proteins between PBMC and purified immune cell types, as well as changing patterns of overlapping expression for PBMC and each cell type at each time point after TIV vaccination. Substantial variability was observed in the number of cell type-specific DE proteins, with less than 20% being shared between both subjects for most cell types and time points (S8 Table. Shared DE proteins; S8 Dataset. Shared up-regulated DE proteins; and S9 Dataset. Shared down-regulated DE proteins). Similar to transcriptomic data, semi-supervised hierarchical clustering revealed little overlap in the shared DE proteins from each cell type at each time point after TIV vaccination (Fig. 7A-C).

**Fig 8:**
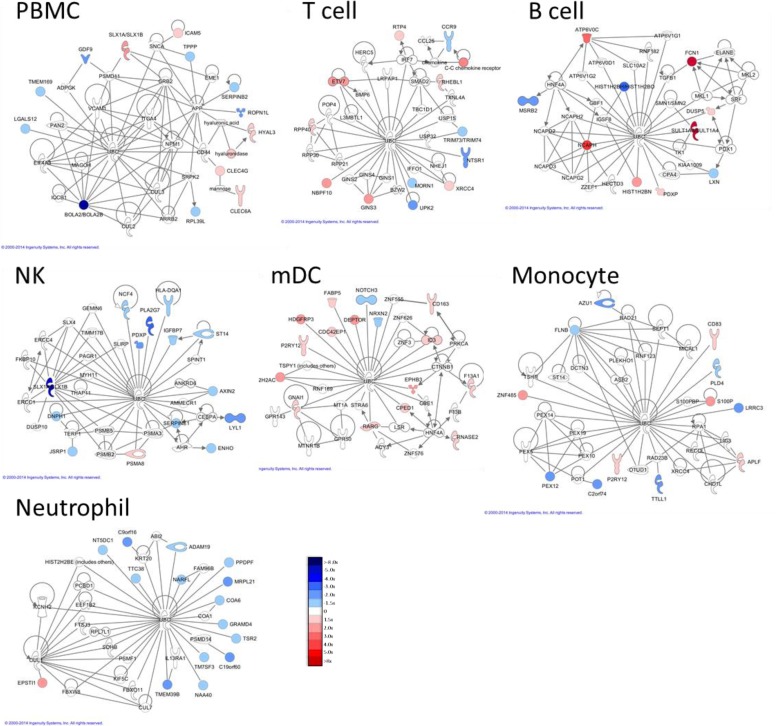
Networks derived from DE RNA transcripts at d1 post-TIV vaccination. Differentially expressed protein-coding RNA transcripts (1.5x, p<0.05) identified in both TIV-vaccinated subjects at day 1 post-vaccination were imported into IPA, and the top network identified in each cell type is displayed. Very little overlap of individual transcripts or biological networks that are activated is observed between cell types.

**Fig 9:**
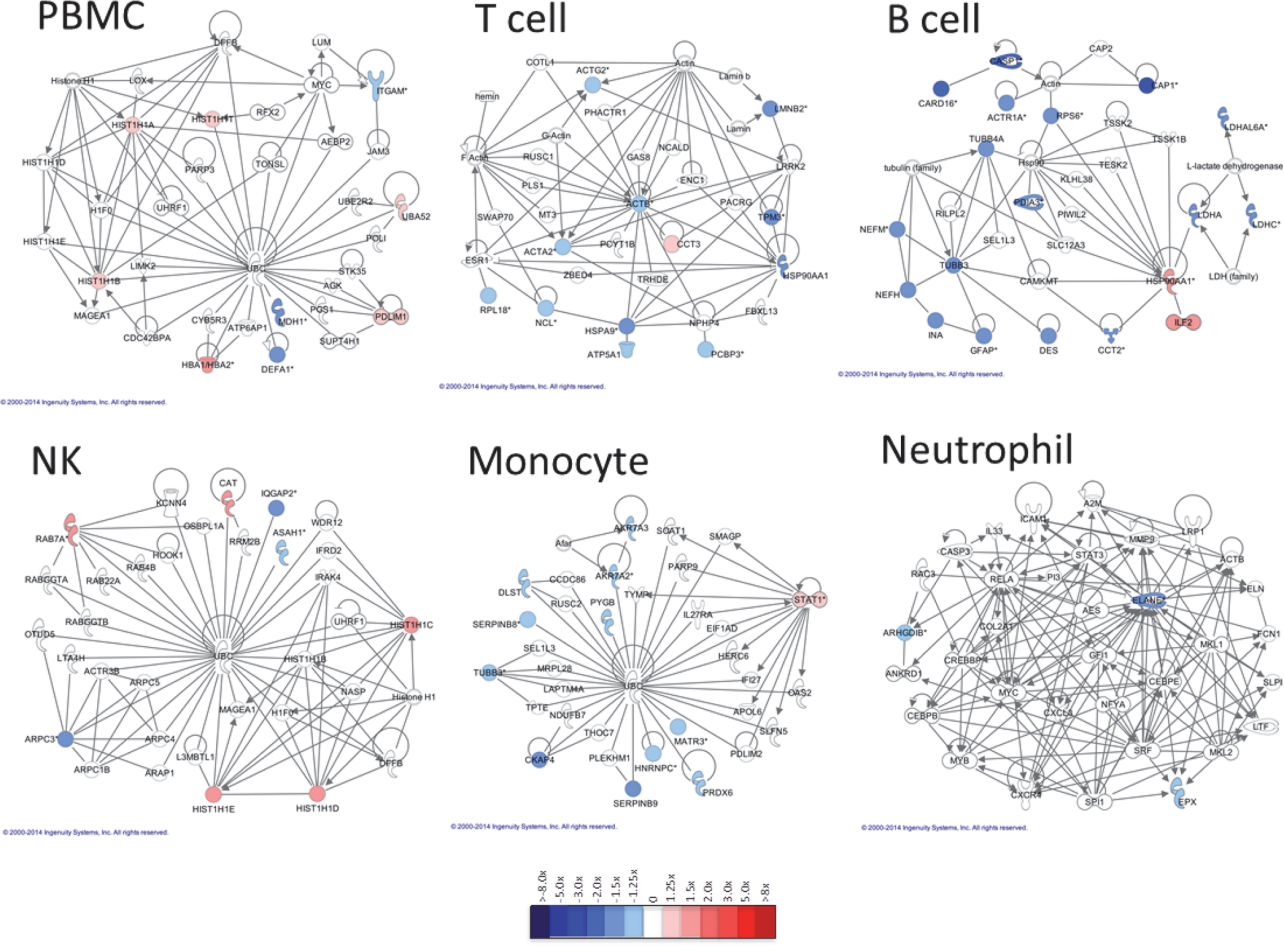
Networks derived from DE proteins at d1 post-TIV vaccination. Differentially expressed proteins (1.25x) identified in both TIV-vaccinated subjects at day 1 post-vaccination were imported into IPA, and the top network identified in each cell type is displayed (*multiple ENSPs mapped to these proteins). Very little overlap of individual proteins or biological networks that are activated is observed between cell types. B cell data was derived from only one subject due to insufficient recovery of B cells from the second subject.

Following cluster analysis, lists of shared DE transcripts or proteins from each cell type and time point were loaded into *Ingenuity Pathway Analysis* (*IPA)* to identify the most significant biological interactions after TIV vaccination. When comparing the top network identified in each cell type for both protein-coding RNA transcripts and proteins (Figs. 8 and 9, respectively), each cell type induced unique biological networks at day 1 after TIV vaccination. Similarly, unique RNA and protein networks were observed in each cell type at day 3 and day 7 after vaccination (S11 Fig. Networks derived from DE RNA transcripts at d3 post-TIV vaccination; S12 Fig. Networks derived from DE RNA transcripts at d7 post-TIV vaccination; S13 Fig. Networks derived from DE proteins at d3 post-TIV vaccination; and S14 Fig. Networks derived from DE proteins at d7 post-TIV vaccination). The top biological networks and canonical pathways identified in each cell type at each time point are shown in S10 Dataset. Top networks and pathways identified in TIV-vaccinated subjects.

## Discussion

The goal of this study was to develop methods and establish protocols that can be used in future systems vaccinology studies. By utilizing this efficient cell-sorting protocol, we obtained sufficient numbers of six immune cell types purified from freshly collected whole blood to perform both RNA-sequencing and quantitative proteomics experiments. Importantly, cells were processed and stored for downstream applications in a single day, thus avoiding the pitfalls of freeze-thaw cycles on downstream analysis. In this study, sorting was stopped once the target number of cells was reached (1.5–3 x10^6^ cells) even if MACS-enriched material remained. Collection of larger numbers of cells is therefore possible for some cell types. In this regard, we have utilized this protocol in a subsequent vaccinology study and collected up to 4x10^6^ neutrophils, up to 3x10^6^ B cells, T cells, NK and monocytes, and up to 1x10^6^ mDC from similar amounts of starting material.

Further fractionation of these six cell types into sub-populations was considered. However, we decided against this approach for several reasons. First, we were interested in broadly sampling the immune system in response to vaccination. Previous vaccinology studies that investigated responses of individual immune cells only focused on selected cell types [9,10]. Our approach profiled both transcriptomic and quantitative proteomic responses of six essential innate and adaptive immune cell types, including neutrophils and NK cells, after vaccination. Signals from small, potentially important sub-populations from any of these immune cell types may still be masked in our systems analysis. However, by sorting for these six immune cell types, we simultaneously investigated both innate and adaptive immune cell responses to vaccination at a cell-specific level. Second, pursuing sub-populations of immune cells would require either obtaining larger blood samples or reducing the number of distinct cell types that we could purify in order to recover sufficient cells for both RNA-Seq and proteomics analyses. If only transcriptomic studies had been performed, sorting for sub-populations from selected immune cell types would have been possible. Finally, the added cost for analysis of both transcriptomic and proteomic data from additional sub-populations was considered prohibitive for this study’s broad survey of innate and adaptive immune responses after vaccination. Future studies that focus on a specific immune cell type(s) and/or sub-populations can easily be performed by adapting our protocol, especially if only transcriptomic analysis is proposed.

Emerging technologies that allow for greater identification of sub-populations of cells and the potential for single cell analysis are now possible [45]. For example, CyTOF offers an opportunity to investigate both cell surface and intracellular protein expression at the single cell level [46]. This technology allows for staining of a potentially unlimited number of cellular markers by eliminating the spectral overlap that plagues traditional flow cytometry applications due to use of fluorescently-labeled antibodies. Therefore, analysis of a substantially increased number of cell subtypes from a single sample can be performed. However, the destructive nature of this technology (single cell ICP mass cytometry) eliminates the potential to collect live cells for further downstream applications. Additionally, by nature, antibody-targeted validation studies require that previously identified molecules be selected for screening. The approach described in this study generated both unbiased and quantitative global transcriptomic and proteomic data from six purified immune cell populations after vaccination. CyTOF offers a powerful, single-cell, high throughput approach to validate and characterize results derived by these types of systems studies.

This study optimized our strategy to generate and analyze RNA-seq and quantitative proteomics data from individual immune cell types sorted from fresh human blood. Differential analysis for each immune cell type revealed unique transcriptomic and proteomic expression profiles as well as changing biological networks during the early response after vaccination. Lending support to our strategy, previous transcriptional findings from systems analysis after TIV vaccination were identified in our approach. For example, we found that B cell-specific transcripts identified by Nakaya *et al*. as correlative predictors of protective immunity following TIV vaccination, including immunoglobulin genes and TNFSFR17 as well as the transcription factor XBP-1, were up-regulated in sorted B cell samples from both of our subjects 7 days after vaccination (S1 Dataset)[9]. Additionally, we found that CXCR3, the receptor for CXCL10/IP-10, was significantly up-regulated in both PBMC and sorted B cell samples after TIV vaccination (S1 Dataset); CXCL10/IP-10 was the only cytokine Nakaya *et al*. identified as being significantly increased in the serum of TIV-vaccinated subjects in their systems study[9]. These data suggest that our subject cohort likely attained at least some measure of protection after TIV vaccination. Future studies using these protocols will correlate vaccine-induced differential expression of both RNA and proteins, as well as serum cytokine levels, with day 28 antibody titers to make predictions about generation of protective immunity in response to vaccination.

The methods and strategies developed in this project provided a unique and important opportunity to investigate the quantitative and qualitative differences between PBMC and individual immune cell types at both the transcriptomic and proteomic levels. By utilizing RNA-seq rather than microarray analysis, we were able to identify and quantify an expanded fraction of the transcriptome, which included 29 different classes of RNA transcripts. Additionally, both transcriptomic and proteomic data were visualized across the human reference genome sequence. Only a small fraction of differentially expressed transcripts and proteins identified in the purified immune cell types were also identified in the PBMC fraction. Thus, by analyzing each cell type individually, cell-specific transcriptomic and proteomic contributions to the immune response following vaccination were identified. This cell type-specific information, coupled with unbiased systems biology approaches, provides a more comprehensive approach to monitor and eventually model vaccine responses. The approaches developed in this pilot project will help to guide future systems biology studies aimed at modeling and predicting complex responses to vaccines and vaccine adjuvants involving interactions between multiple cell types.

## Supporting Information

S1 Dataset RNA-seq quality control. (XLSX)

S2 Dataset Normalized transcript expression in human immune cells prior to and post-TIV vaccination. (XLSX)

S3 Dataset Normalized transcript expression in human immune cells filtered for an RPKM of 1.0 in at least one sample from one subject. (XLSX)

S4 Dataset Normalized protein expression in human immune cells prior to and post-TIV vaccination. (XLSX)

S5 Dataset Normalized protein expression in human immune cells filtered to remove zero values and contaminating keratins from one subject. (XLSX)

S6 Dataset Shared up-regulated DE RNA transcripts. (XLSX)

S7 Dataset Shared down-regulated DE RNA transcripts. (XLSX)

S8 Dataset Shared up-regulated DE proteins. (XLSX)

S9 Dataset Shared down-regulated DE proteins. (XLSX)

S10 Dataset Top networks and pathways identified in TIV-vaccinated subjects. (XLSX)

S1 Fig RNA quality control. Scatter plots showing the correlation of total RNA transcripts between time points and subjects. **(a)** Time point comparison within the same subject (HD30 PBMC day 3 vs HD30 PBMC day 0). **(b)** Subject-to-subject comparison of one time point (HD30 PBMC day 3 vs HD31 PBMC day 3). Both comparisons show correlation greater than 0.95.
(TIF)

S2 Fig Proteomics quality control. **(a)** Scatter plot showing the protein abundances measured in two technical replicates of the ICCS common control. Each dot represents an individual protein. X axis represents the protein abundance measured in replicate 2. Y-axis represents the protein abundances measured in replicate 1. **(b)** Scatter plot showing the distribution of fold changes of proteins with respect to their abundances. Each dot represents an individual protein. X axis represents protein abundance. Y axis represents fold changes. **(c)** Cluster dot plot showing the distribution of fold changes in different iTRAQ channels. Each dot represents an individual protein and the lines represent patterns of expression change.
(TIF)

S3 Fig Flow chart for immune cell purification. **(a)** When 150–300x10^6^ PBMC were obtained, B cells (CD19^+^), monocytes (CD14^+^) and T cells (CD3^+^) were first positively selected from the PBMC fraction by MACS; approximately 15% of PBMC were dedicated for CD3^+^ enrichment, 35% of PBMC were dedicated to CD14^+^ enrichment, and 45% of PBMC were dedicated to CD19^+^ enrichment. Negative flow through material was collected, pooled and subsequently depleted of remaining CD3^+^, CD14^+^, CD15^+^, and CD19^+^ cells to enrich for mDC and NK cells. All MACS enriched cell populations were stained as in Fig. 1A with the addition of 7-AAD for live/dead cell identification and subjected to FACS sorting to yield highly purified cell populations. **(b)** When >300x10^6^ PBMC were obtained, CD3+, CD19+ and CD14+ selection was performed as in (a), with a smaller cell fraction dedicated to each sort, while NK and mDC were enriched by negative selection directly from PBMC. Cells were stained and FACS sorted as in (a). **(c)** When <150x10^6^ PBMC were obtained, all PBMC were dedicated to CD19^+^ B cell selection. The CD19-negative flow through was then subjected to CD3^+^CD14^+^ dual positive selection. MACS enriched cells were stained as in (a), and B cells were FACS sorted from the CD19^+^ fraction, T cells and monocytes were FACS sorted from the CD3^+^CD14^+^ fraction, and NK and mDC were FACS sorted from the CD19^-^CD3^-^CD14^-^ fraction. Any potential contaminating neutrophils were eliminated from the NK and mDC fraction by staining with anti-CD15 during FACS sorting.
(TIF)

S4 Fig Individual cell types are not activated by the sorting process. Aliquots of whole blood (WB), PBMC and pooled sorted cells (∼10,000 each cell type) from a representative subject were stained with antibodies directed against CD3, CD11c, CD14, CD15, CD19 and CD56 for phenotyping as in Fig. 1A, as well as CD69, CD86 and CD134 to measure cellular activation. Fluorescence minus one (FMO) controls were used to determine background fluorescence levels for activation marker staining in each cell type from WB and PBMC samples. Assessment of surface expression (mean fluorescence intensity; MFI) of **(a)** CD69 in each cell type, **(b)** CD86 in monocyes, B cells, and mDC, and **(c)** CD134 in T cells reveals that none of the cell types were significantly activated during any step of our sorting protocol.
(TIF)

S5 Fig Adequate RNA quantity and quality is obtained from sorted immune cells for RNA-seq applications. RNA isolated from sorted immune cells (500,000 each cell type except mDC, which contained 400,000 at d0, 567,000 at d1, 438,000 at d3, and 548,000 at d7) from a single vaccinated subject was quantified (top panel) and evaluated for RNA integrity (bottom panel) as described in Materials and Methods.
(TIF)

S6 Fig Transcriptional profiling of PBMC and individual immune cell types. Baseline, day 0 RNA profiles of PBMC and each purified cell type (all transcript classes represented, non-zero transcripts with an RPKM of 1 in at least one sample; ∼21,000 transcripts) from a single subject were plotted using Circos to visualize relative expression of transcripts across the genome. Bars on the outside of the circle represent individual chromosomes. The heat-map color scaling parameter was set to "scale_log_base = 1" to allow for optimal color space.
(TIF)

S7 Fig Adequate protein quantity is obtained from sorted immune cells for proteomics applications. Total protein isolated from sorted immune cells (1x10^6^ each cell type) from a single vaccinated subject was quantified as described in Materials and Methods.
(TIF)

S8 Fig Two iTRAQ strategies for quantitative proteomic analysis of immune cells after vaccination. **(a)** Experimental design. In strategy 1, multiple immune cell types from one time point were multiplexed together in the experiment. In strategy 2, different time points from the same immune cell type were multiplexed together. An immune cell common standard (ICCS) was used to normalize reporter ion intensities across the experiments. **(b)** Unsupervised hierarchical clustering analysis and **(c)** PCA of pseudo-spectral counts from one subject generated using strategy 1 (left panels; 5,676 proteins, filtered to remove zero values and contaminating keratins) or strategy 2 (right panels, 3,852 proteins, filtered to remove zero values and contaminating keratins) reveals that cell-types cluster together and display distinct cell-type specific patterns of protein expression using strategy 2, but not with strategy 1.
(TIF)

S9 Fig Proteomic profiling of PBMC and individual immune cell types. Baseline, day 0 protein profiles of PBMC and each purified cell type (3,852 proteins) from a single subject were plotted using Circos to visualize relative expression of proteins across the genome. Bars on the outside of the circle represent individual chromosomes. The heat-map color scaling parameter was set to "scale_log_base = 10" to allow for optimal color space.
(TIF)

S10 Fig Principal component analysis reveals poor correlation of proteomes between subjects. **(a)** RNA transcripts (all RNA classes represented, filtered to remove zero values; ∼39,106 total transcripts) and **(b)** proteins (5,304 total proteins, filtered to remove zero values and contaminating keratins) from subject 1 (HD31; large circles) and subject 2 (HD30; small circles) were clustered in the same experiment. RNA from both subjects clusters similarly, while proteins do not.
(TIF)

S11 Fig Networks derived from DE RNA transcripts at d3 post-TIV vaccination. Differentially expressed protein-coding RNA transcripts (1.5x, *p*<0.05) identified in both TIV-vaccinated subjects at day 3 post-vaccination were imported into *IPA*, and the top network identified in each cell type is displayed. Very little overlap of individual transcripts or biological networks that are activated is observed between cell types.
(TIF)

S12 Fig Networks derived from DE RNA transcripts at d7 post-TIV vaccination. Differentially expressed protein-coding RNA transcripts (1.5x, *p*<0.05) identified in both TIV-vaccinated subjects at day 7 post-vaccination were imported into *IPA*, and the top network identified in each cell type is displayed. Very little overlap of individual transcripts or biological networks that are activated is observed between cell types.
(TIF)

S13 Fig Networks derived from DE proteins at d3 post-TIV vaccination. Differentially expressed proteins (1.25x) identified in both TIV-vaccinated subjects at day 3 post-vaccination were imported into *IPA*, and the top network identified in each cell type is displayed (*multiple ENSPs mapped to these proteins). Very little overlap of individual proteins or biological networks that are activated is observed between cell types. B cell data was derived from only one subject due to insufficient recovery of B cells from the second subject.
(TIF)

S14 Fig Networks derived from DE proteins at d7 post-TIV vaccination. Differentially expressed proteins (1.25x) identified in both TIV-vaccinated donors at day 7 post-vaccination were imported into *IPA*, and the top network identified in each cell type is displayed (*multiple ENSPs mapped to these proteins). Very little overlap of individual proteins or biological networks that are activated is observed between cell types. B cell data was derived from only one subject due to insufficient recovery of B cells from the second subject.
(TIF)

S1 Table Summary of baseline RNA transcripts identified in each cell type from one subject by RNA-seq analysis. (TIF)

S2 Table Comparison of differentially expressed RNA transcripts in PMBC and individual immune cell types. (TIF)

S3 Table Shared DE RNA transcripts (all transcript classes represented). (TIF)

S4 Table Total splicing events identified in each cell type. (TIF)

S5 Table Differential splicing events identified in each subject, cell type and time point. (TIF)

S6 Table Shared differential splicing. (TIF)

S7 Table Comparison of differentially expressed proteins in PMBC and individual immune cell types. (TIF)

S8 Table Shared DE proteins. (TIF)
