# Correction: A Cell-Based Systems Biology Assessment of Human Blood to Monitor Immune Responses after Influenza Vaccination

**DOI:** 10.1371/journal.pone.0122550

**Published:** 2015-03-30

**Authors:** The PLOS ONE Staff

There are errors in the Funding section. The correct funding information is as follows: This project was funded in part with Federal funds from the National Institutes of Allergy and Infectious Disease, National Institutes of Health, Department of Health and Human Services, under Contract No. 272200800007C, CTSA award No. UL1TR000445 from the National Center for Advancing Translational Sciences, the Childhood Infections Research Program grant T32-AI095202-01, the Immunobiology of Blood and Vascular Systems training grant 5 T32 HL69765-12, and NIH grant GM064779. The contents of this manuscript are solely the responsibility of the authors and do not necessarily represent official views of the National Center for Advancing Translational Sciences or the National Institutes of Health. The funders had no role in the study design, data collection and analysis, decision to publish, or preparation of the manuscript.
