# Supplementary figures and images for: A Cell-Based Systems Biology Assessment of Human Blood to Monitor Immune Responses after Influenza Vaccination
Source: PLoS One. 2015 Feb 23;10(2):e0118528. doi: 10.1371/journal.pone.0118528 (PMC4338067; doi:10.1371/journal.pone.0118528)

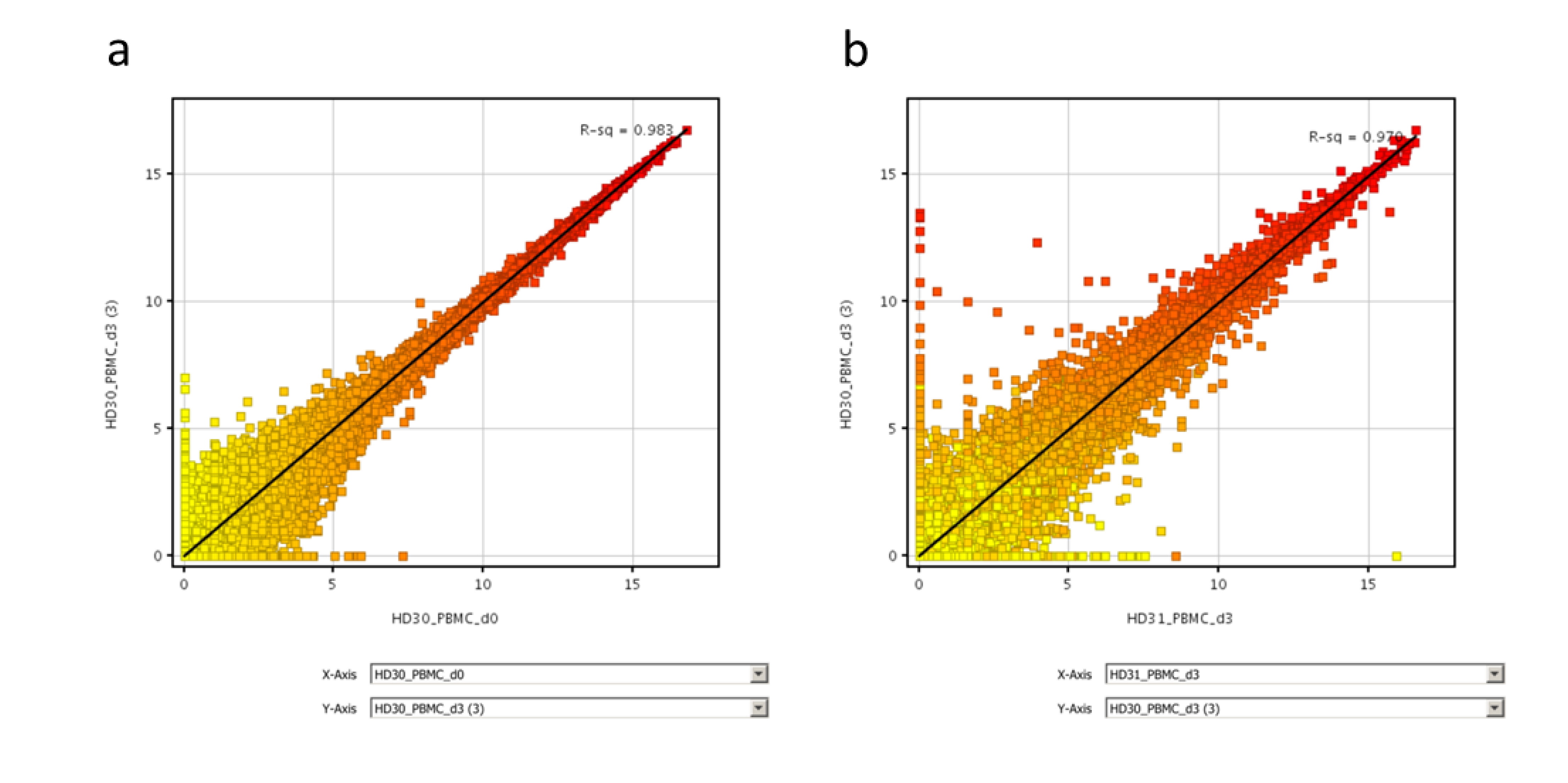

Supplement: S1 Fig — Scatter plots showing the correlation of total RNA transcripts between time points and subjects. (a) Time point comparison within the same subject (HD30 PBMC day 3 vs HD30 PBMC day 0). (b) Subject-to-subject comparison of one time point (HD30 PBMC day 3 vs HD31 PBMC day 3). Both comparisons show correlation greater than 0.95. (TIF) [file pone.0118528.s011.tif]

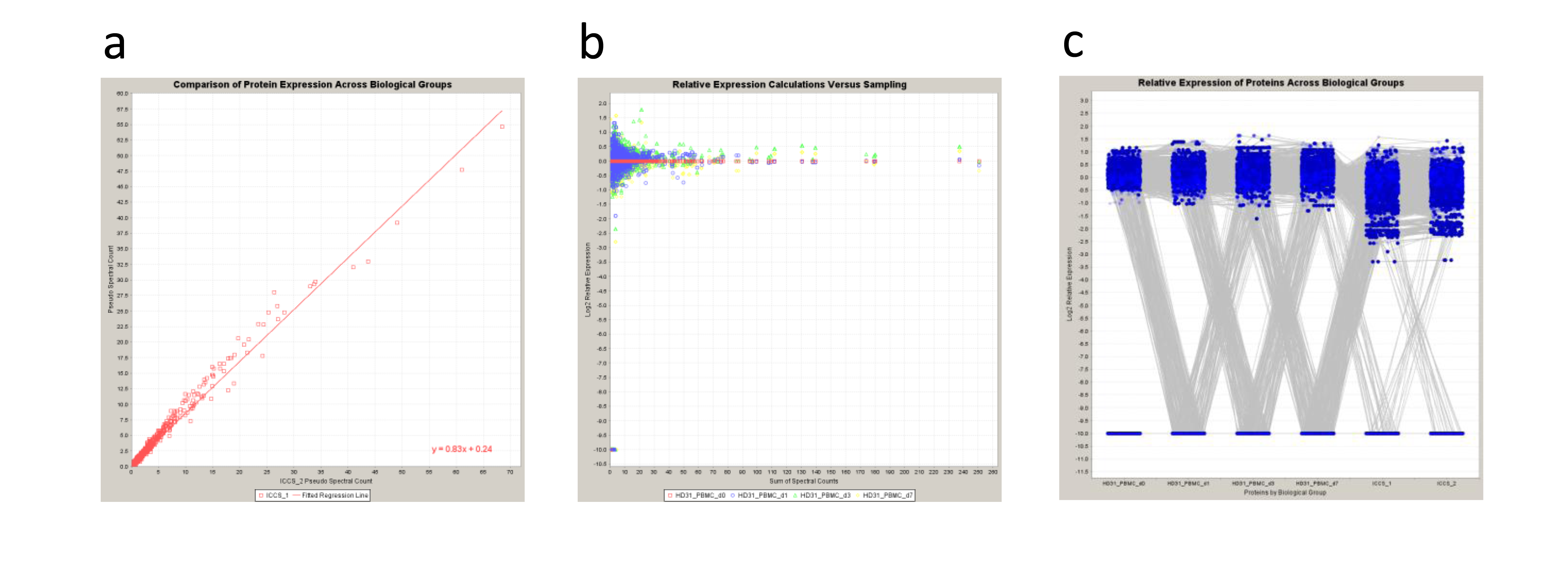

Supplement: S2 Fig — (a) Scatter plot showing the protein abundances measured in two technical replicates of the ICCS common control. Each dot represents an individual protein. X axis represents the protein abundance measured in replicate 2. Y-axis represents the protein abundances measured in replicate 1. (b) Scatter plot showing the distribution of fold changes of proteins with respect to their abundances. Each dot represents an individual protein. X axis represents protein abundance. Y axis represents fold changes. (c) Cluster dot plot showing the distribution of fold changes in different iTRAQ channels. Each dot represents an individual protein and the lines represent patterns of expression change. (TIF) [file pone.0118528.s012.tif]

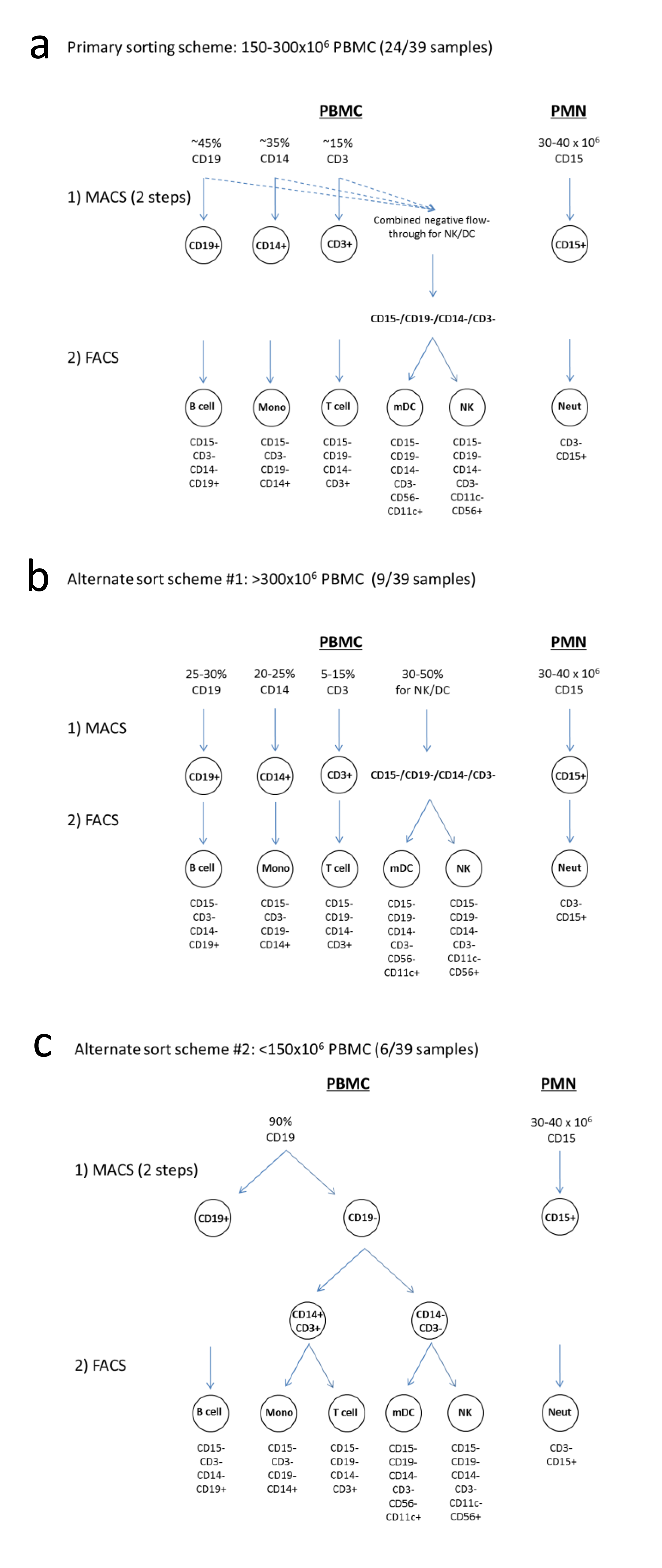

Supplement: S3 Fig — (a) When 150–300x106 PBMC were obtained, B cells (CD19+), monocytes (CD14+) and T cells (CD3+) were first positively selected from the PBMC fraction by MACS; approximately 15% of PBMC were dedicated for CD3+ enrichment, 35% of PBMC were dedicated to CD14+ enrichment, and 45% of PBMC were dedicated to CD19+ enrichment. Negative flow through material was collected, pooled and subsequently depleted of remaining CD3+, CD14+, CD15+, and CD19+ cells to enrich for mDC and NK cells. All MACS enriched cell populations were stained as in Fig. 1A with the addition of 7-AAD for live/dead cell identification and subjected to FACS sorting to yield highly purified cell populations. (b) When >300x106 PBMC were obtained, CD3+, CD19+ and CD14+ selection was performed as in (a), with a smaller cell fraction dedicated to each sort, while NK and mDC were enriched by negative selection directly from PBMC. Cells were stained and FACS sorted as in (a). (c) When <150x106 PBMC were obtained, all PBMC were dedicated to CD19+ B cell selection. The CD19-negative flow through was then subjected to CD3+CD14+ dual positive selection. MACS enriched cells were stained as in (a), and B cells were FACS sorted from the CD19+ fraction, T cells and monocytes were FACS sorted from the CD3+CD14+ fraction, and NK and mDC were FACS sorted from the CD19-CD3-CD14- fraction. Any potential contaminating neutrophils were eliminated from the NK and mDC fraction by staining with anti-CD15 during FACS sorting. (TIF) [file pone.0118528.s013.tif]

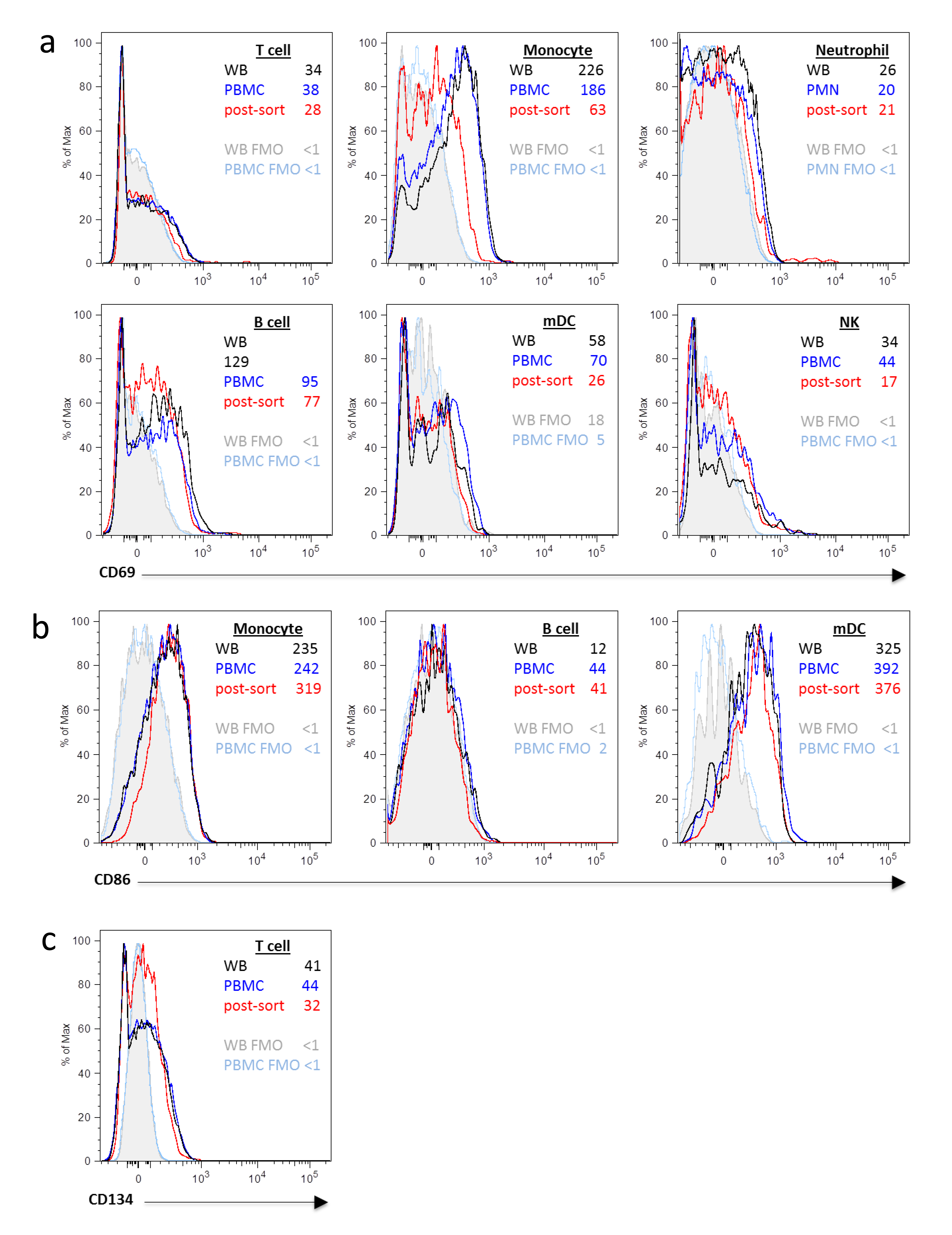

Supplement: S4 Fig — Aliquots of whole blood (WB), PBMC and pooled sorted cells (∼10,000 each cell type) from a representative subject were stained with antibodies directed against CD3, CD11c, CD14, CD15, CD19 and CD56 for phenotyping as in Fig. 1A, as well as CD69, CD86 and CD134 to measure cellular activation. Fluorescence minus one (FMO) controls were used to determine background fluorescence levels for activation marker staining in each cell type from WB and PBMC samples. Assessment of surface expression (mean fluorescence intensity; MFI) of (a) CD69 in each cell type, (b) CD86 in monocyes, B cells, and mDC, and (c) CD134 in T cells reveals that none of the cell types were significantly activated during any step of our sorting protocol. (TIF) [file pone.0118528.s014.tif]

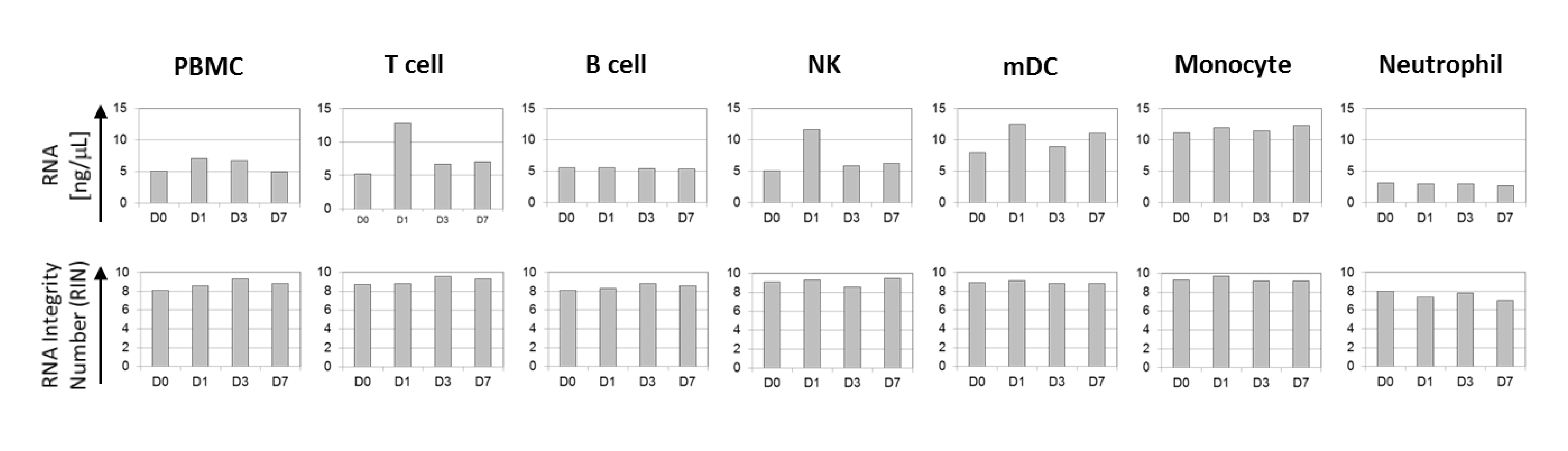

Supplement: S5 Fig — RNA isolated from sorted immune cells (500,000 each cell type except mDC, which contained 400,000 at d0, 567,000 at d1, 438,000 at d3, and 548,000 at d7) from a single vaccinated subject was quantified (top panel) and evaluated for RNA integrity (bottom panel) as described in Materials and Methods. (TIF) [file pone.0118528.s015.tif]

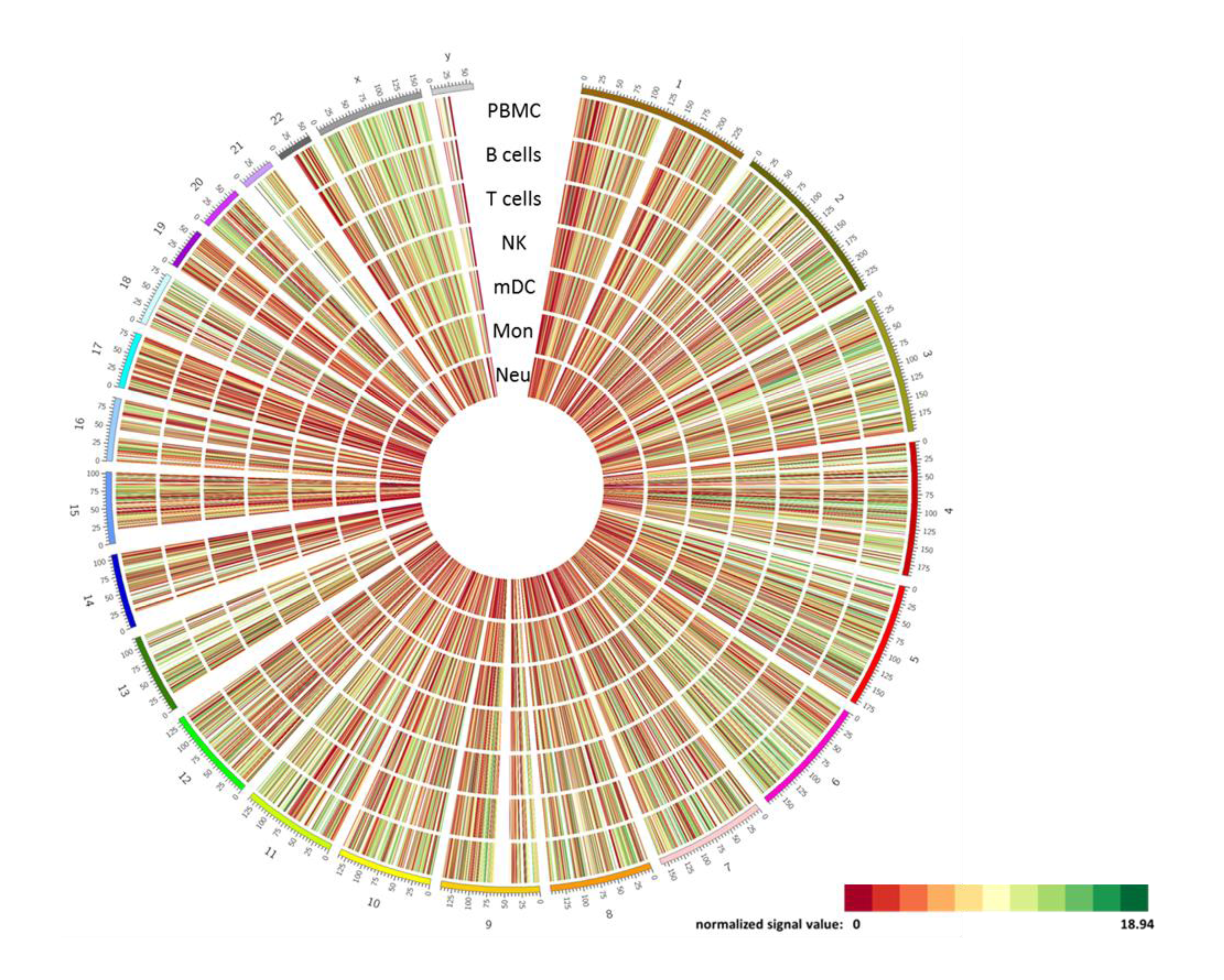

Supplement: S6 Fig — Baseline, day 0 RNA profiles of PBMC and each purified cell type (all transcript classes represented, non-zero transcripts with an RPKM of 1 in at least one sample; ∼21,000 transcripts) from a single subject were plotted using Circos to visualize relative expression of transcripts across the genome. Bars on the outside of the circle represent individual chromosomes. The heat-map color scaling parameter was set to "scale_log_base = 1" to allow for optimal color space. (TIF) [file pone.0118528.s016.tif]

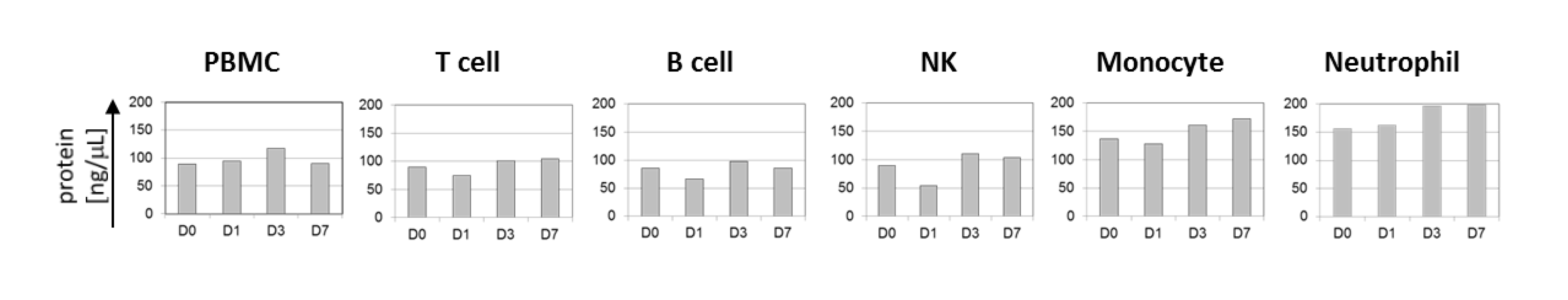

Supplement: S7 Fig — Total protein isolated from sorted immune cells (1x10^6^ each cell type) from a single vaccinated subject was quantified as described in Materials and Methods. (TIF) [file pone.0118528.s017.tif]

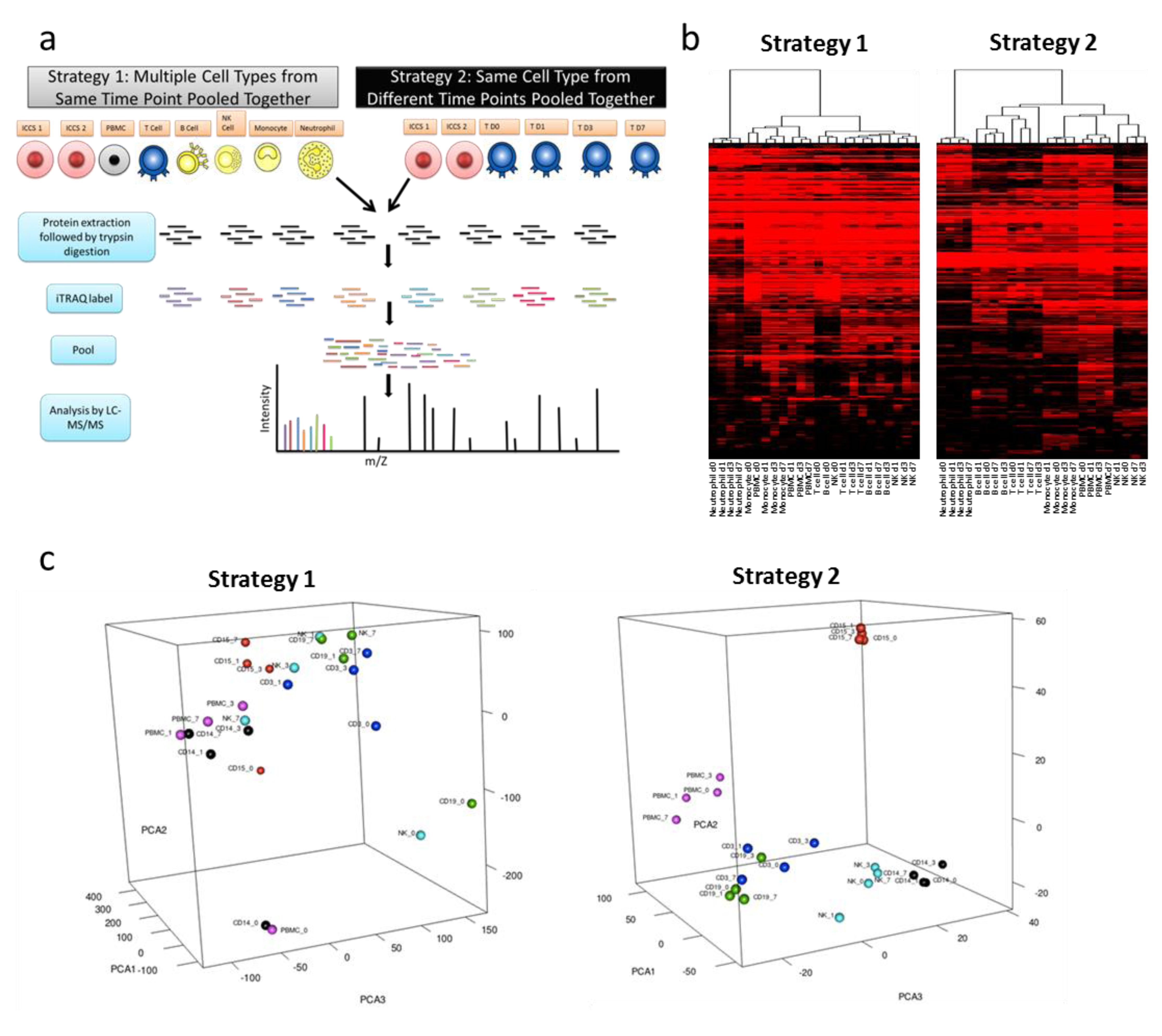

Supplement: S8 Fig — (a) Experimental design. In strategy 1, multiple immune cell types from one time point were multiplexed together in the experiment. In strategy 2, different time points from the same immune cell type were multiplexed together. An immune cell common standard (ICCS) was used to normalize reporter ion intensities across the experiments. (b) Unsupervised hierarchical clustering analysis and (c) PCA of pseudo-spectral counts from one subject generated using strategy 1 (left panels; 5,676 proteins, filtered to remove zero values and contaminating keratins) or strategy 2 (right panels, 3,852 proteins, filtered to remove zero values and contaminating keratins) reveals that cell-types cluster together and display distinct cell-type specific patterns of protein expression using strategy 2, but not with strategy 1. (TIF) [file pone.0118528.s018.tif]

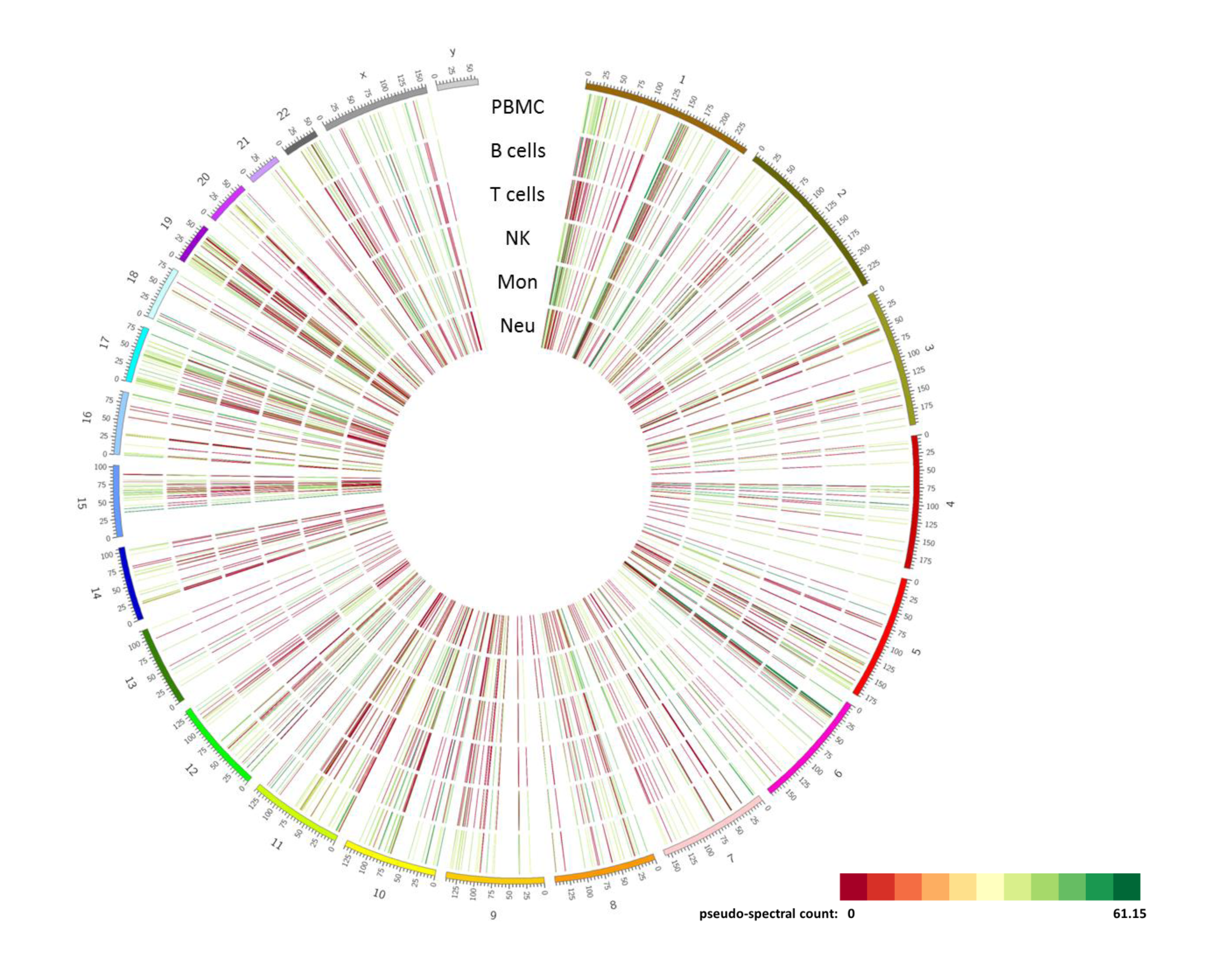

Supplement: S9 Fig — Baseline, day 0 protein profiles of PBMC and each purified cell type (3,852 proteins) from a single subject were plotted using Circos to visualize relative expression of proteins across the genome. Bars on the outside of the circle represent individual chromosomes. The heat-map color scaling parameter was set to "scale_log_base = 10" to allow for optimal color space. (TIF) [file pone.0118528.s019.tif]

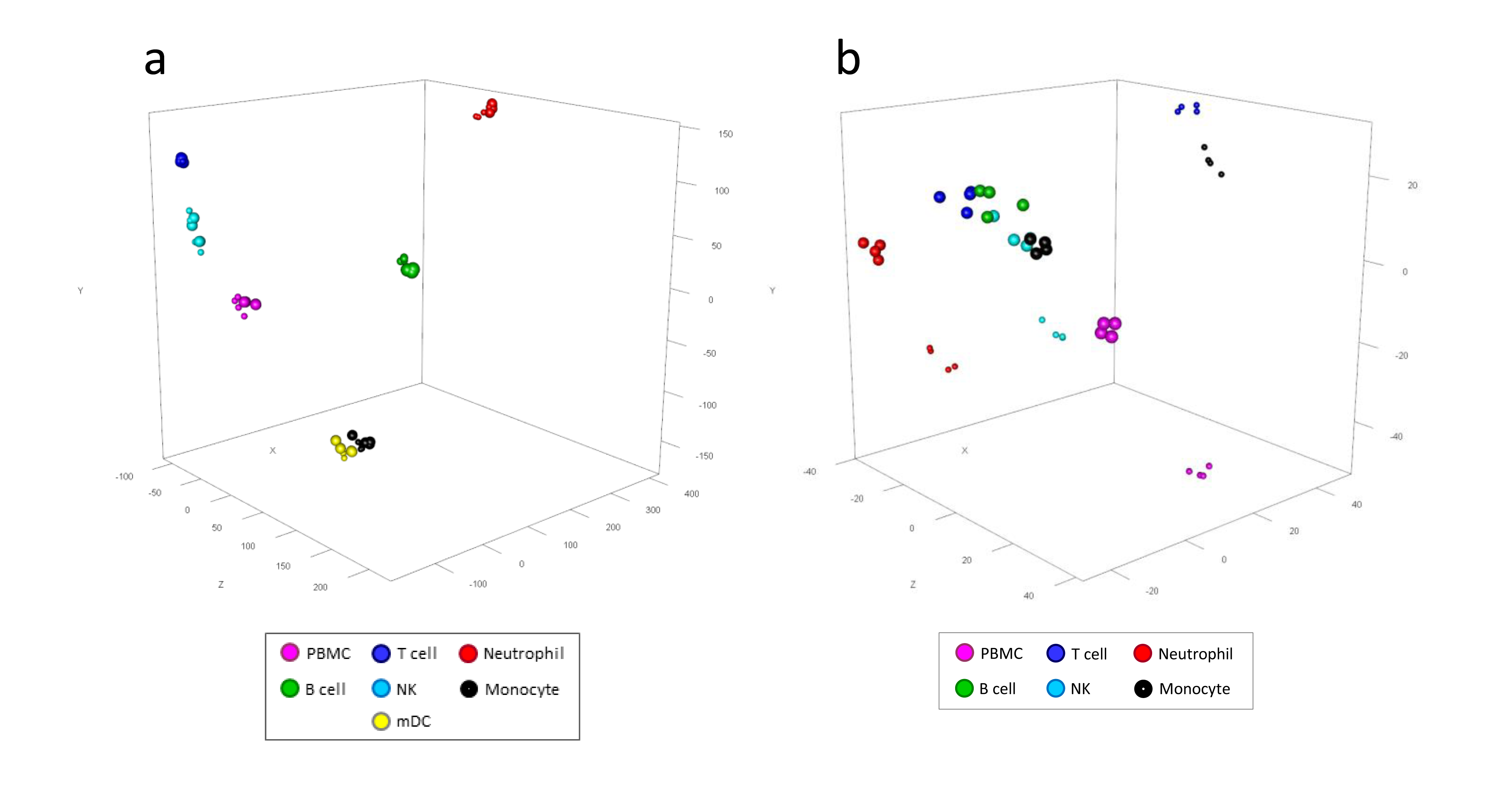

Supplement: S10 Fig — (a) RNA transcripts (all RNA classes represented, filtered to remove zero values; ∼39,106 total transcripts) and (b) proteins (5,304 total proteins, filtered to remove zero values and contaminating keratins) from subject 1 (HD31; large circles) and subject 2 (HD30; small circles) were clustered in the same experiment. RNA from both subjects clusters similarly, while proteins do not. (TIF) [file pone.0118528.s020.tif]

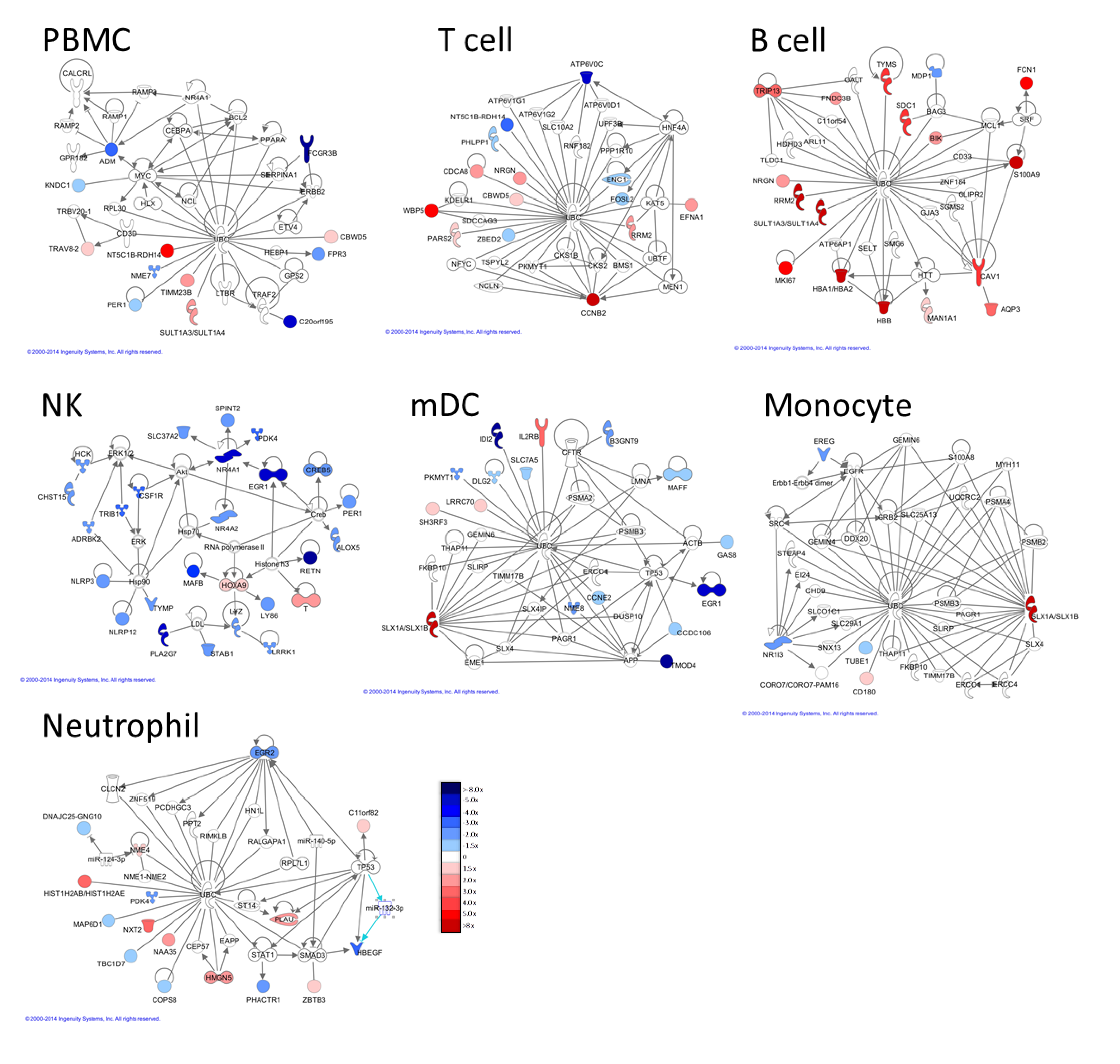

Supplement: S11 Fig — Differentially expressed protein-coding RNA transcripts (1.5x, p<0.05) identified in both TIV-vaccinated subjects at day 3 post-vaccination were imported into IPA, and the top network identified in each cell type is displayed. Very little overlap of individual transcripts or biological networks that are activated is observed between cell types. (TIF) [file pone.0118528.s021.tif]

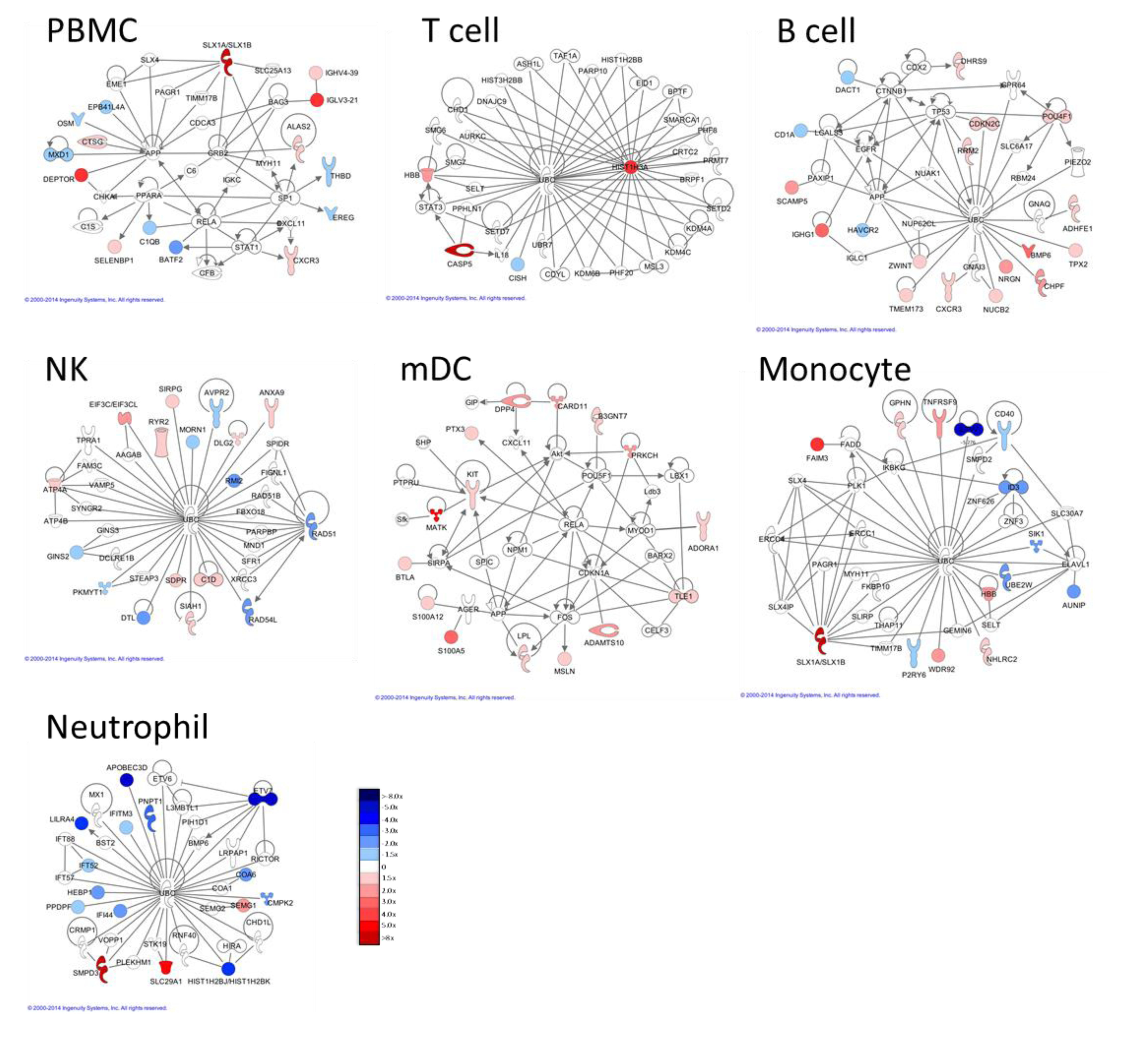

Supplement: S12 Fig — Differentially expressed protein-coding RNA transcripts (1.5x, p<0.05) identified in both TIV-vaccinated subjects at day 7 post-vaccination were imported into IPA, and the top network identified in each cell type is displayed. Very little overlap of individual transcripts or biological networks that are activated is observed between cell types. (TIF) [file pone.0118528.s022.tif]

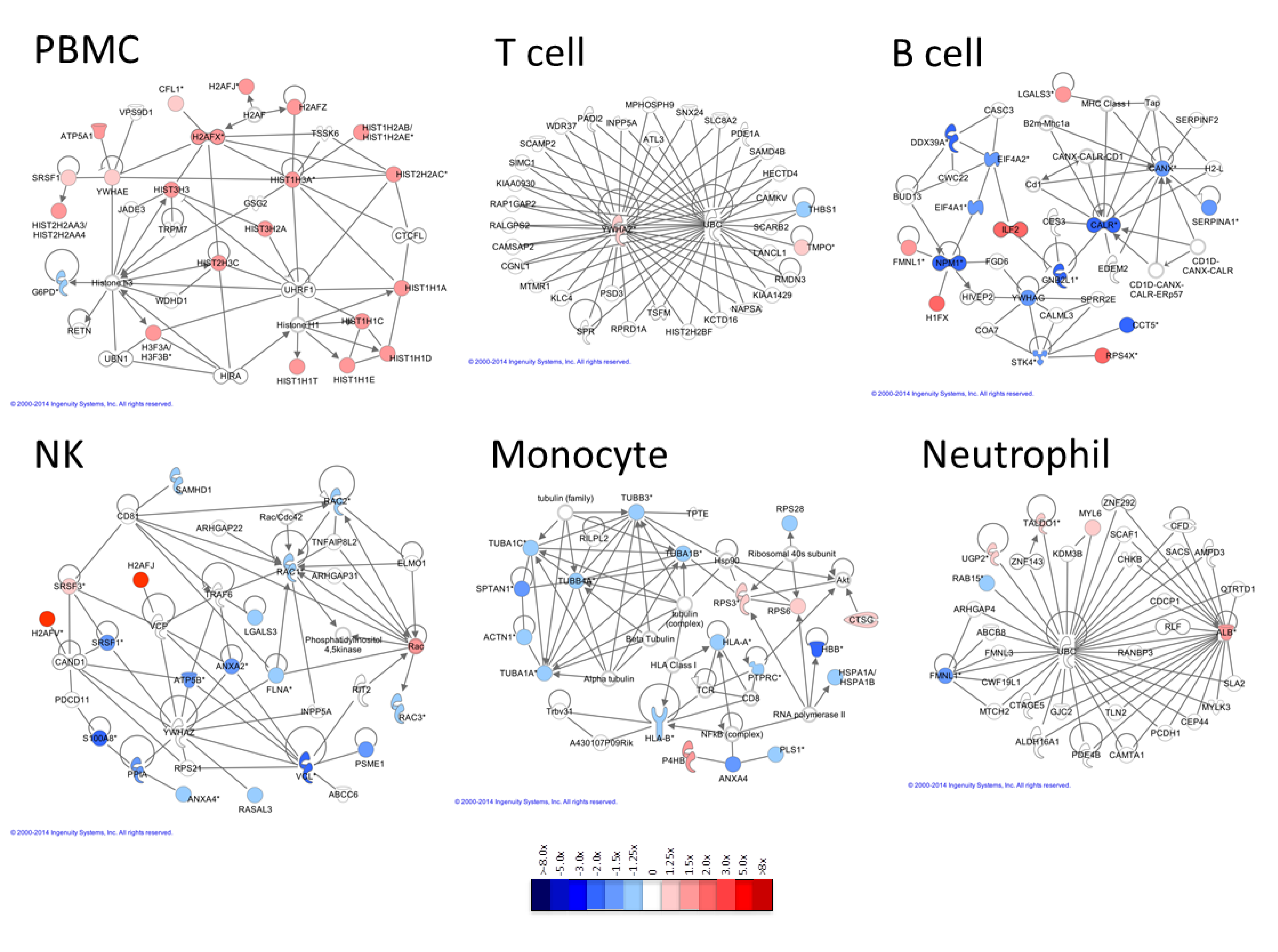

Supplement: S13 Fig — Differentially expressed proteins (1.25x) identified in both TIV-vaccinated subjects at day 3 post-vaccination were imported into IPA, and the top network identified in each cell type is displayed (*multiple ENSPs mapped to these proteins). Very little overlap of individual proteins or biological networks that are activated is observed between cell types. B cell data was derived from only one subject due to insufficient recovery of B cells from the second subject. (TIF) [file pone.0118528.s023.tif]

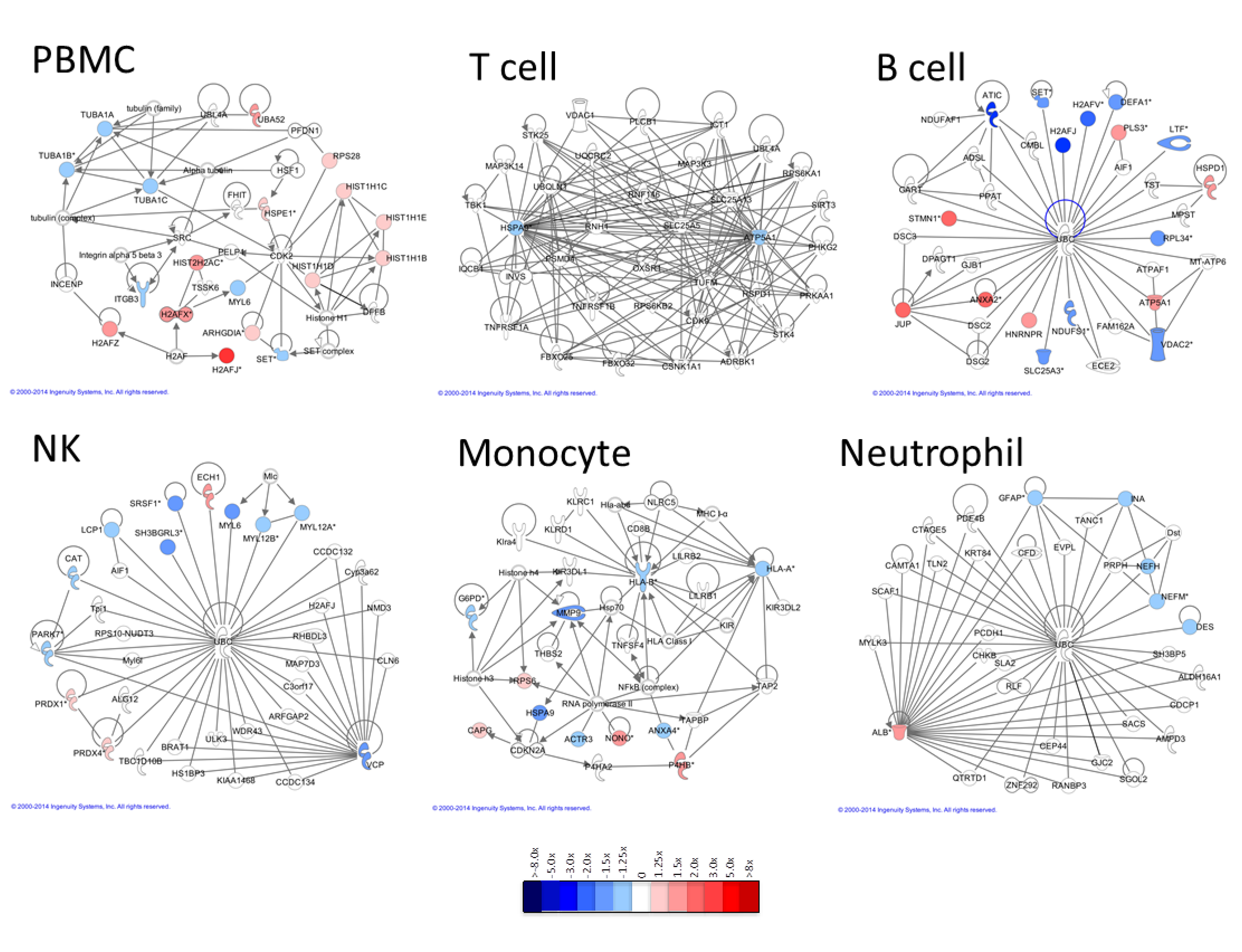

Supplement: S14 Fig — Differentially expressed proteins (1.25x) identified in both TIV-vaccinated donors at day 7 post-vaccination were imported into IPA, and the top network identified in each cell type is displayed (*multiple ENSPs mapped to these proteins). Very little overlap of individual proteins or biological networks that are activated is observed between cell types. B cell data was derived from only one subject due to insufficient recovery of B cells from the second subject. (TIF) [file pone.0118528.s024.tif]

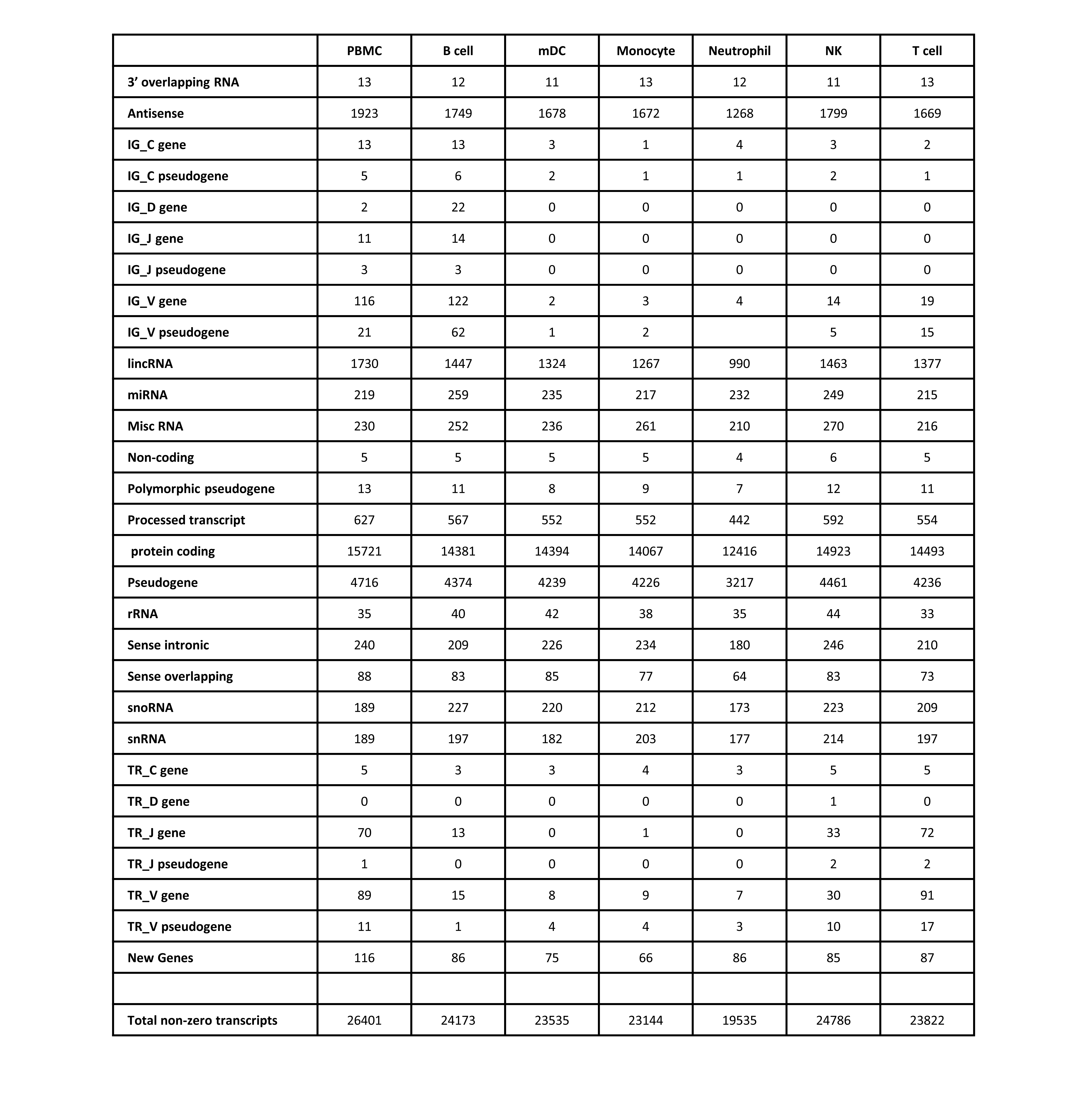

Supplement: S1 Table — (TIF) [file pone.0118528.s025.tif]

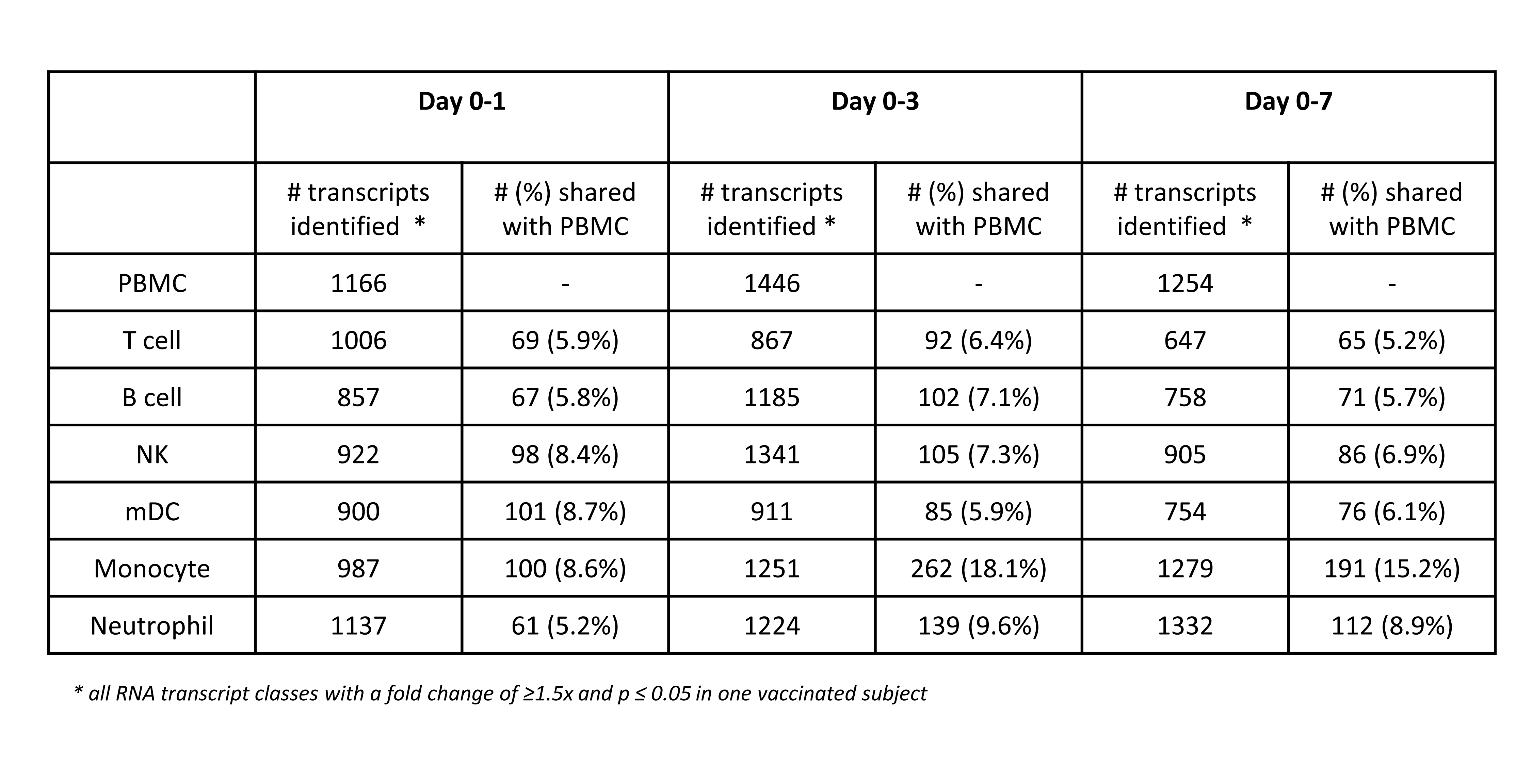

Supplement: S2 Table — (TIF) [file pone.0118528.s026.tif]

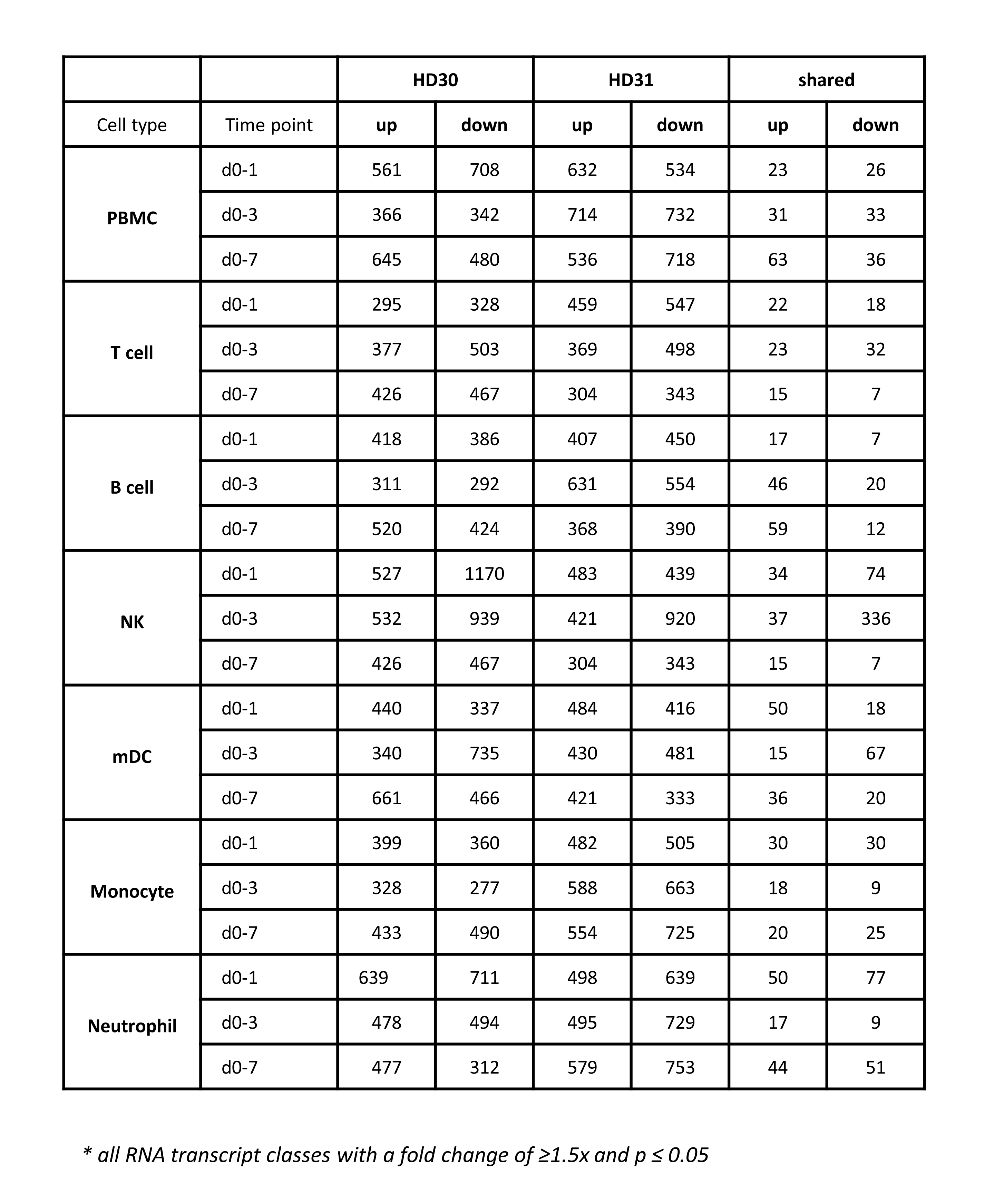

Supplement: S3 Table — (TIF) [file pone.0118528.s027.tif]

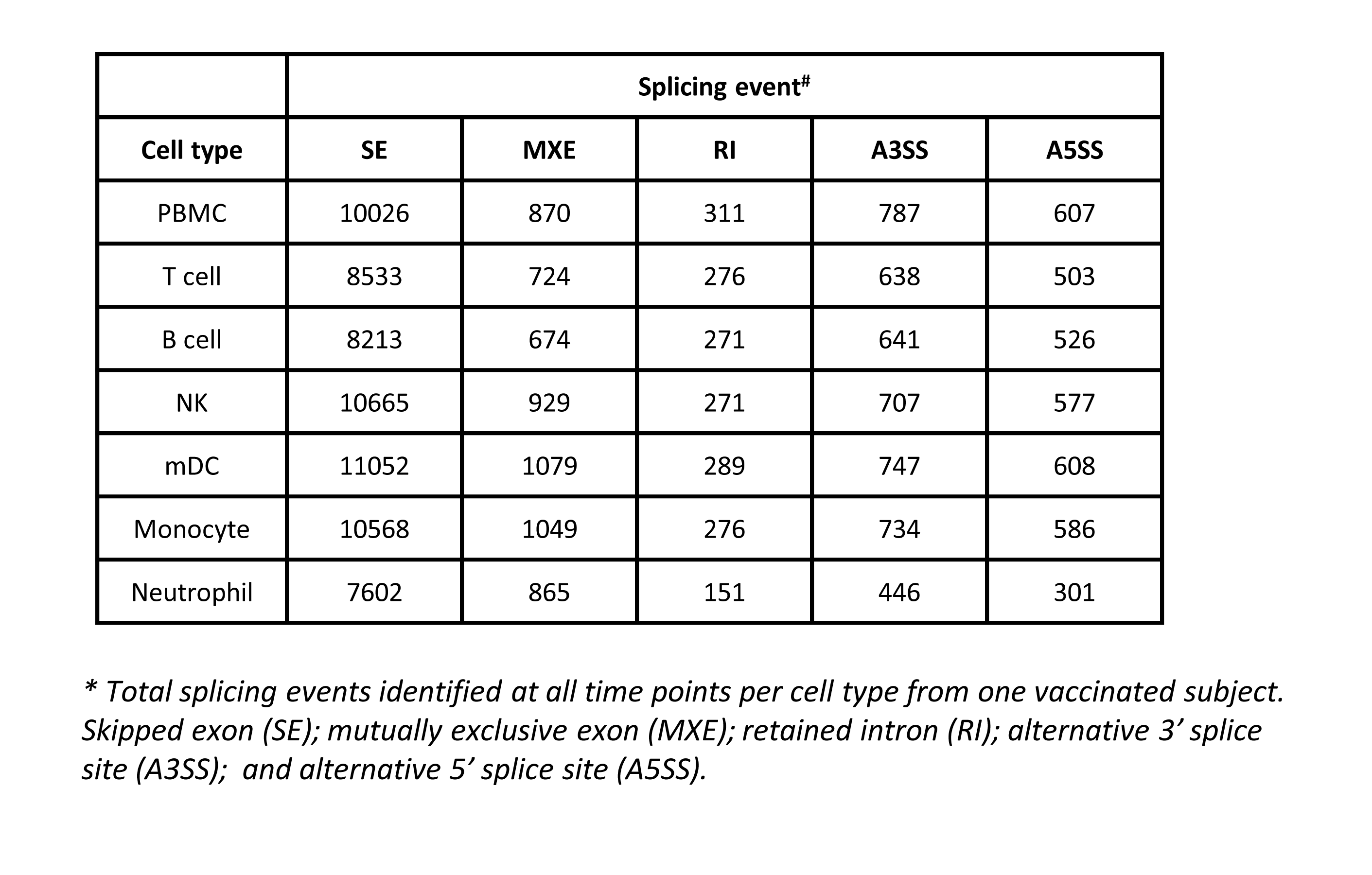

Supplement: S4 Table — (TIF) [file pone.0118528.s028.tif]

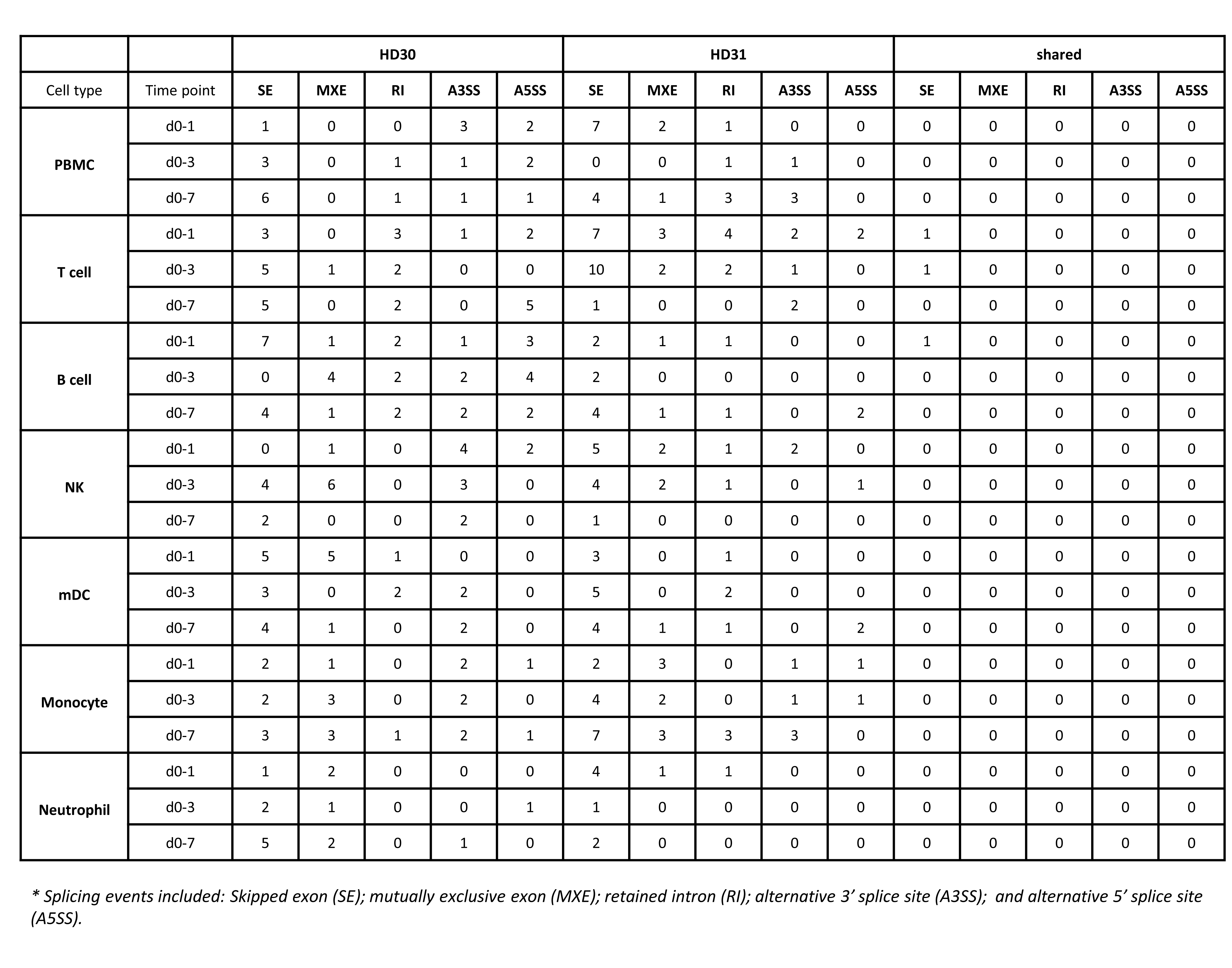

Supplement: S5 Table — (TIF) [file pone.0118528.s029.tif]

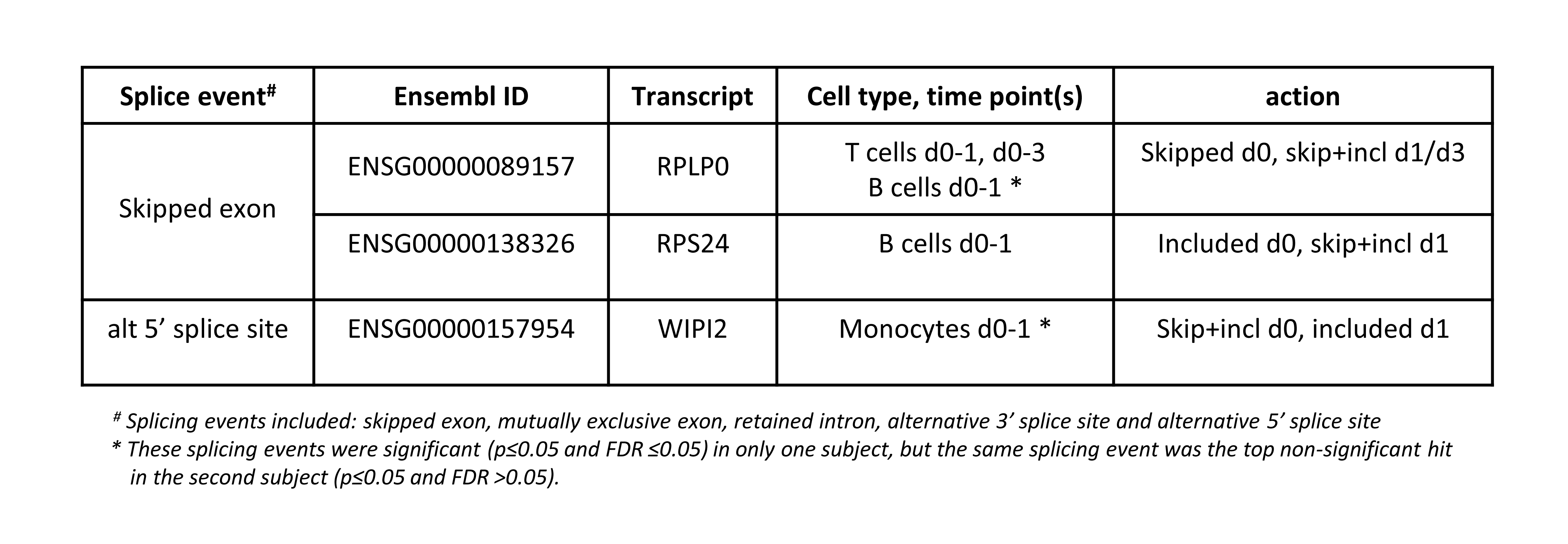

Supplement: S6 Table — (TIF) [file pone.0118528.s030.tif]

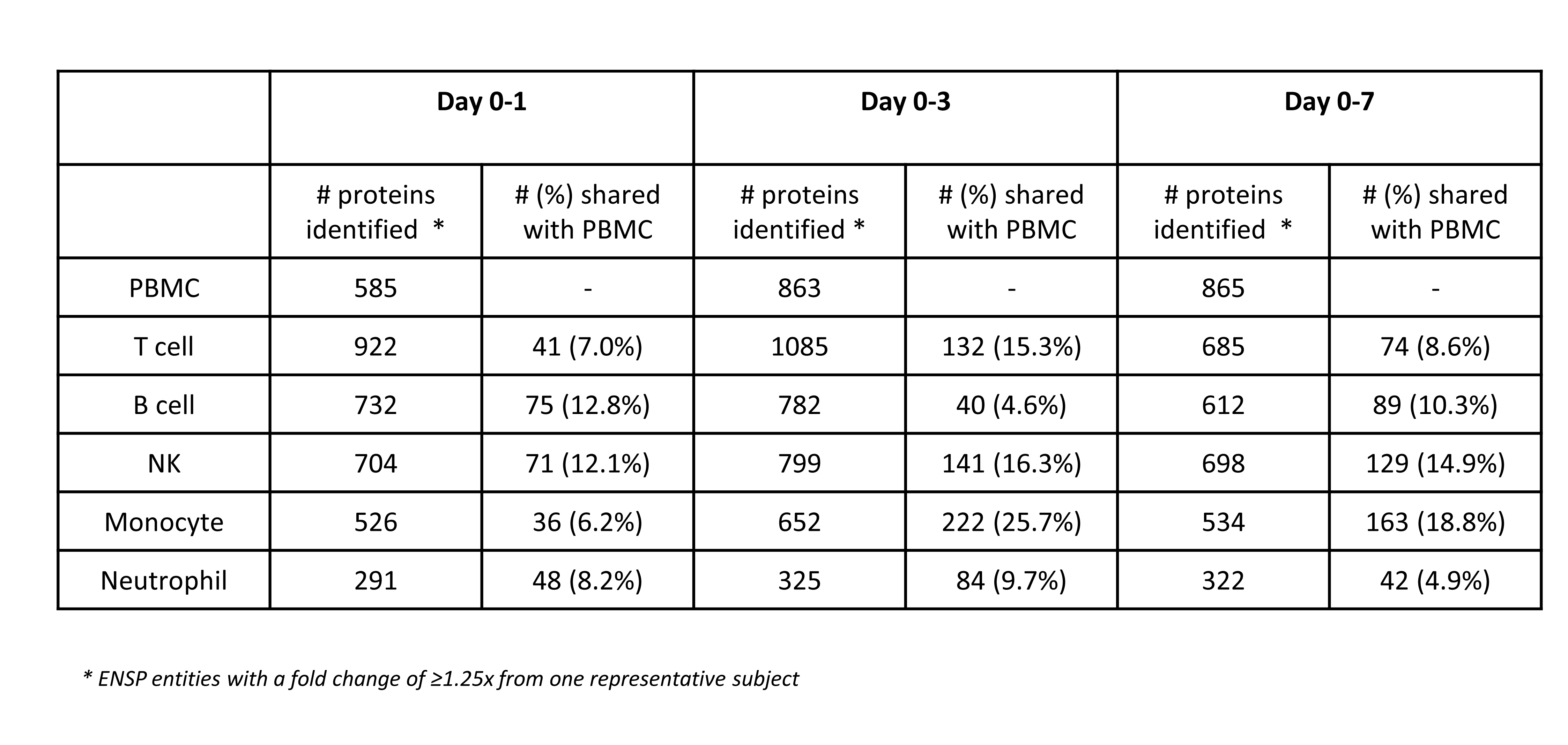

Supplement: S7 Table — (TIF) [file pone.0118528.s031.tif]

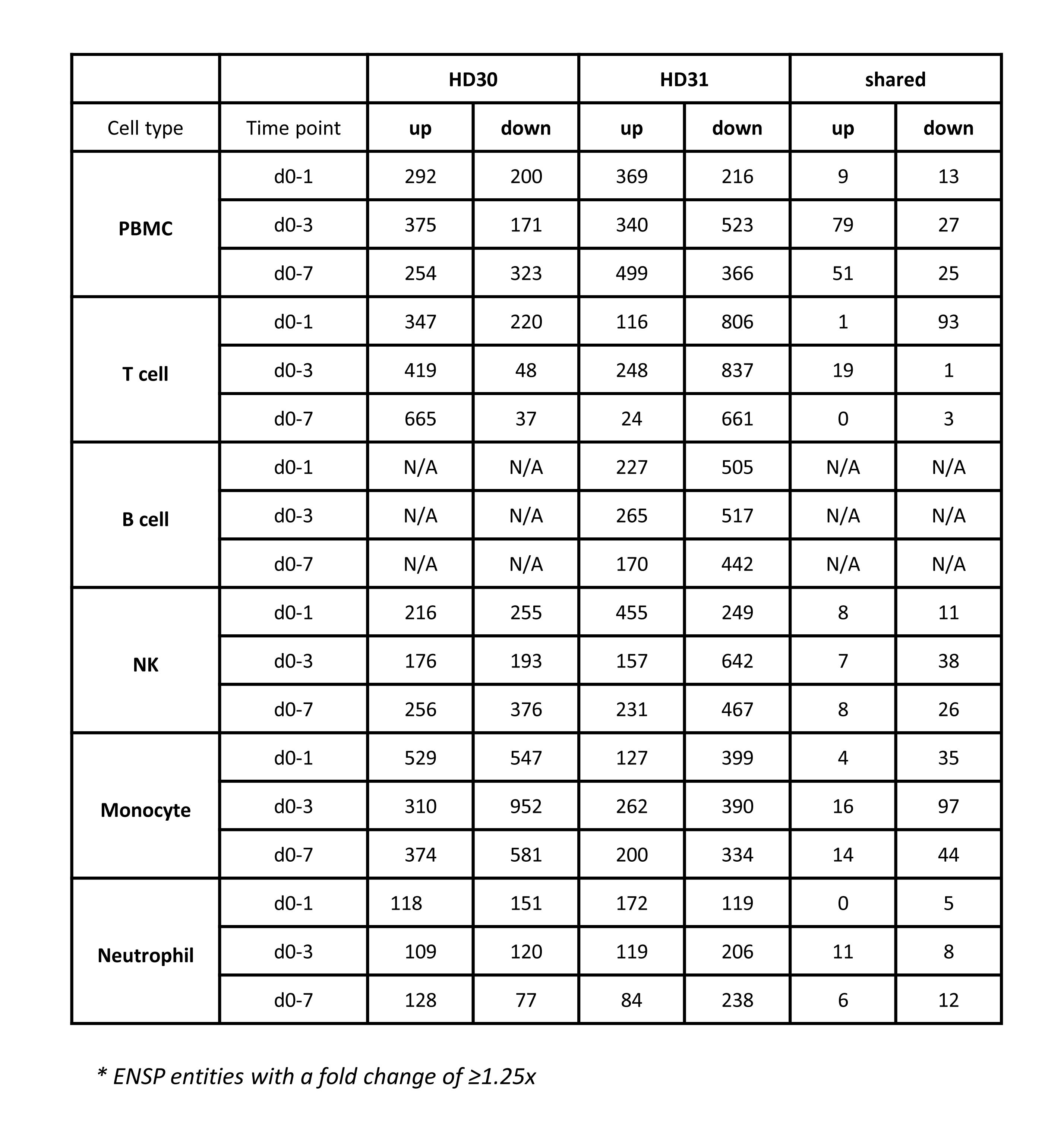

Supplement: S8 Table — (TIF) [file pone.0118528.s032.tif]
